# The Complete Genome Sequence of a Second Distinct Betabaculovirus from the True Armyworm, *Mythimna unipuncta*

**DOI:** 10.1371/journal.pone.0170510

**Published:** 2017-01-19

**Authors:** Robert L. Harrison, Daniel L. Rowley, Joseph Mowery, Gary R. Bauchan, David A. Theilmann, George F. Rohrmann, Martin A. Erlandson

**Affiliations:** 1 Invasive Insect Biocontrol and Behavior Laboratory, Beltsville Agricultural Research Center, USDA Agricultural Research Service, Beltsville, Maryland, United States of America; 2 Electron and Confocal Microscopy Unit, Beltsville Agricultural Research Center, USDA Agricultural Research Service, Beltsville, Maryland, United States of America; 3 Summerland Research and Development Centre, Agriculture and Agri-Food Canada, Summerland, British Columbia, Canada; 4 Department of Microbiology, Oregon State University, Corvallis, Oregon, United States of America; 5 Saskatoon Research and Development Centre, Agriculture and Agri-Food Canada, Saskatoon, Saskatchewan, Canada; Agriculture and Agri-Food Canada, CANADA

## Abstract

The betabaculovirus originally called Pseudaletia (Mythimna) sp. granulovirus #8 (MyspGV#8) was examined by electron microscopy, host barcoding PCR, and determination of the nucleotide sequence of its genome. Scanning and transmission electron microscopy revealed that the occlusion bodies of MyspGV#8 possessed the characteristic size range and morphology of betabaculovirus granules. Barcoding PCR using cytochrome oxidase I primers with DNA from the MyspGV#8 collection sample confirmed that it had been isolated from the true armyworm, *Mythimna unipuncta* (Lepidoptera: Noctuidae) and therefore was renamed MyunGV#8. The MyunGV#8 genome was found to be 144,673 bp in size with a nucleotide distribution of 49.9% G+C, which was significantly smaller and more GC-rich than the genome of Pseudaletia unipuncta granulovirus H (PsunGV-H), another *M*. *unipuncta* betabaculovirus. A phylogeny based on concatenated baculovirus core gene amino acid sequence alignments placed MyunGV#8 in clade *a* of genus *Betabaculovirus*. Kimura-2-parameter nucleotide distances suggested that MyunGV#8 represents a virus species different and distinct from other species of *Betabaculovirus*. Among the 153 ORFs annotated in the MyunGV#8 genome, four ORFs appeared to have been obtained from or donated to the alphabaculovirus lineage represented by Leucania separata nucleopolyhedrovirus AH1 (LeseNPV-AH1) during co-infection of *Mythimna* sp. larvae. A set of 33 ORFs was identified that appears only in other clade *a* betabaculovirus isolates. This clade *a*-specific set includes an ORF that encodes a polypeptide sequence containing a CIDE_N domain, which is found in caspase-activated DNAse/DNA fragmentation factor (CAD/DFF) proteins. CAD/DFF proteins are involved in digesting DNA during apoptosis.

## Introduction

Viruses of family *Baculoviridae* possess a double-stranded circular DNA genome that is packaged into enveloped, rod-shaped capsids [1]. These viruses, which have been isolated exclusively from insects, have been shown to produce two virion forms, budded virus (BV) and occlusion-derived virus (ODV) [2]. BVs, which are produced first, acquire an envelope as nucleocapsids bud through the host cell plasma membrane. At later times during replication, nucleocapsids of genera *Alphabaculovirus*, *Gammabaculovirus*, and *Deltabaculovirus* acquire an envelope within the host cell nucleus to form the occlusion-derived virus (ODV). ODVs of genus *Betabaculovirus* obtain their envelope in a nucleo-cytoplasmic milieu formed after breakdown of the host nuclear envelope. ODVs are subsequently assembled into occlusion bodies consisting largely of single, highly expressed viral protein, known as polyhedrin (alpha-, delta- and gammabaculoviruses) or granulin (betabaculoviruses).

Research on baculovirus replication in insect cell lines and on polyhedrin gene expression led to the development of methods and reagents for a recombinant protein expression system using baculoviruses and cell lines [3]. The baculovirus expression vector system (BEVS) has been a very popular option for producing recombinant protein, and inspired an explosion of research on the basic virology and molecular biology of baculoviruses, with a concentration of effort on tractable isolates of baculovirus species such as *Autographa californica multiple nucleopolyhedrovirus*, *Bombyx mori nucleopolyhedrovirus*, and *Orgyia pseudotsugata multiple nucleopolyhedrovirus*.

There has been a long-held interest in baculoviruses as potential biocontrol agents for the management of insect pests [4, 5]. This interest predated the development of the BEVS, and it motivated the field collection and acquisition of many baculovirus isolates by various laboratories beginning in the 1950s and early 1960s. The application of PCR and DNA sequencing methods to characterize baculovirus isolates in collections has greatly expanded our comprehension of the genetic diversity in *Baculoviridae*, especially in genera *Alphabaculovirus* and *Betabaculovirus*. Sequence data from a large number of isolates from collections allowed for the formulation and proposal of baculovirus species demarcation criteria that have been used by other researchers [6].

Recently, the characterization by PCR and sequencing of several baculovirus isolates collected and assembled by Dr. Mauro Martignoni during his career at the USDA Forest Service Laboratory in Corvallis, Oregon, USA was described [7]. Partial sequence data from the *lef-8* gene (encoding a baculovirus RNA polymerase subunit) of 26 isolates from the Martignoni collection indicated that ten of these isolates represented previously uncharacterized baculoviruses. Since then, the complete genome nucleotide sequences for four of these uncharacterized isolates have been determined [8–11].

Among the Martignoni collection isolates that were initially characterized were 6 isolates of viruses listed as being “*Pseudoletia* capsules” or “*Pseudoletia* granulosis”, presumably obtained from larvae of *Mythimna* (formerly *Pseudaletia*) *unipuncta* (true armyworm), a pest of graminaceous crops and pastures. The species *Pseudaletia unipuncta granulovirus* was created by the International Committee of Taxonomy of Viruses (ICTV) in 2002, apparently based on an isolate, Pseudaletia unipuncta granulovirus Hawaiian (PsunGV-H), originally described from Hawaiian populations of *M*. *unipuncta* in 1959 [12]. The sequence of an enhancin gene cloned from PsunGV-H was reported in 1995 [13]. A 2008 study [14] reported partial sequences of *granulin*, *lef-8* and *lef-9* genes amplified from PsunGV-H. A GenBank entry (EU678671) for the complete genome sequence of what appears to be the same virus isolate (Pseudaletia unipuncta granulovirus strain Hawaiin[sic]) was deposited in 2008, though there is no publication on the analysis of the sequence. This genome sequence shares 100% nucleotide sequence identity with the previously reported PsunGV-H *enhancin*, *granulin*, *lef-8*, and *lef-9* sequences. A later study [15] reported partial *granulin*, *lef-8*, and, *lef-9* sequences from a betabaculovirus isolated from infected *M*. *unipuncta* larvae in a hay field in Kentucky, USA. This isolate, MyunGV-KY410, appeared to be a variant of PsunGV-H on the basis of sequence alignment and phylogeny. In contrast, the Martignoni collection *Mythimna* sp. GV isolates appear to differ significantly from PsunGV-H, with *lef-8* nucleotide sequences sharing only 72% identity [7].

To clarify the relationship between the Martignoni collection *Mythimna* sp. betabaculovirus isolates and previously characterized *M*. *unipuncta* betabaculovirus isolates, additional studies focusing on isolate Pseudaletia (Mythimna) sp. granulovirus #8 (MyspGV#8) [7] were carried out. Electron microscopy confirmed that it was a betabaculovirus and DNA barcoding showed that the host insect was *Mythimna unipuncta*. The name of the isolate was therefore revised to Mythimna unipuncta granulovirus #8 (MyunGV#8). The complete genome nucleotide sequence of MyunGV#8 was determined and analyzed. The results indicate that MyunGV#8 represents a second betabaculovirus species infecting *M*. *unipuncta*, and points to a set of genes that define a lineage within genus *Betabaculovirus* that encompasses several species.

## Materials and Methods

### Virus

The virus sample used in this study was dated April 20, 1964 and described as “Pseudoletia granulosis (dirty) stock”. It was subsequently called Pseudoletia (Mythimna) sp granulovirus #8 (MyspGV#8) [7]. However, since the host species was reassigned to genus *Mythimna* and DNA barcoding confirmed the host is *M*. *unipuncta* (see below), the virus was renamed MyunGV#8.

### Electron microscopy

Prior to electron microscopy, an aliquot of this virus was passed through three layers of cheesecloth and washed first with 0.5% SDS, then with 0.1% SDS, and finally with 0.5 M NaCl before being re-suspended in ddH_2_O, as previously described [16].

For scanning electron microscopy (SEM), MyunGV#8 granules were observed in the LT-SEM as described in Bolton et al. (2014) [17].

For transmission electron microscopy (TEM), an aliquot of the granule suspension was centrifuged at 2300x g for 3 min to form a pellet. The pellet was fixed for 2 hours at room temperature in 2.5% glutaraldehyde-0.05M sodium cacodylate-0.005M CaCl_2_ (pH 7.0), and processed for TEM as previously described [18].

### DNA barcoding

A 100 μl aliquot of the MyunGV#8 sample was vortexed briefly, then centrifuged at 5000x g for 5 min. The supernatant was transferred to a fresh 1.5 ml tube. For a positive control, a 3^rd^ instar *M*. *unipuncta* larva was homogenized in 0.1% SDS in a 1.5 mL Eppendorf tube using a blue polypropylene pellet pestle (Sigma-Aldrich, St. Louis, MO). Cuticle fragments were removed from the homogenate and the final volume was brought to 100 μl with deionized distilled H_2_O (ddH_2_O).

DNA was extracted as described by Greenstone et al. (2005) [19] using High Salt Extraction Buffer (0.4 M NaCl-10 mM Tris–HCl pH 8.0–2 mM EDTA pH 8.0) [20] followed by precipitation with isopropanol. DNA was also isolated from 1.5 x 10^6^ Sf9 cells (derived from *Spodoptera frugiperda*; [21]) as described above for use as a negative control.

PCRs for amplification of mitochondrial cytochrome oxidase I (COI) sequences from the template DNAs (1.5 μl/reaction) were set up with Promega GoTaq® DNA Polymerase following the manufacturer’s protocol. Reactions were supplemented with 50 ng PureLink™ RNase A (ThermoFisher Scientific, Waltham, MA, USA) and 0.2% w/v BSA (Sigma-Aldrich, St. Louis, Missouri, USA). Primers used were C1-J-1751 (alias Ron; 5'- GGATCACCTGATATAGCATTCCC-3') and C1-N-2191 (alias Nancy; 5'-CCCGGTAAAATTAAAATATAAACTTC-3') [22]. PCR cycle conditions consisted of an initial denaturation for 3 min at 94.5°C, followed by 40 cycles of 45 s at 94.5°C, 1 min at 39°C, and 2 min at 72°C; 5 min at 72°C completed the program. Amplimers from the reactions were precipitated as previously described [23] and sequenced using BigDye terminator v.3.1 kits on an ABI3130XL Genetic Analyzer (Applied Biosystems, Foster City, California, USA). Assembly of sequence data into contigs, editing, and database searches with contigs was undertaken with Lasergene (DNAStar, Madison, Wisconsin, USA).

### Virus DNA isolation and sequencing

MyunGV#8 granules were solubilized in 0.1 M sodium carbonate and DNA was extracted from occluded virus that had been centrifuged through a 25% w/w sucrose pad, as previously described [24].

DNA sequencing was done at the National Research Council, Plant Biotechnology Institute (Saskatoon, Saskatchewan, Canada) using Roche 454 FLX-titanium pyrosequencing technology. The sequences were assembled using CLC-Genomics Workbench 6.0.2 into an initial contig of 144,510 bp with an average sequence coverage of 170X. PCR and Sanger dideoxy sequencing were carried out to resolve or confirm regions with ambiguous or repeated sequences or unusual features. An additional 163 bp of contiguous sequence was identified, bringing the final assembled genome to 144,673 bp.

The Lasergene SeqManPro (version 12) sequence editor was used to prepare the final contig of the consensus genome sequence. The MyunGV#8 genome sequence generated during this study has been deposited in GenBank with the accession number KX855660.

### ORF and homologous repeat region (*hr*) annotation

The LaserGene GeneQuest program (v. 12; DNASTAR) was used to identify ORFs in the MyunGV#8 genome sequence. ORFs were annotated if they were >50 codons in length and (a) they were evolutionarily conserved with other baculovirus ORFs, as ascertained by BLASTp (e-value <0.010); or (b) they did not overlap a larger ORF by >75 bp and were predicted to be protein-encoding by both the fgenesV (http://linux1.softberry.com/berry.phtml) and ZCURVE_V [25] algorithms. A subset of predicted amino acid sequences were also used in queries on the HHpred server (https://toolkit.tuebingen.mpg.de/hhpred) [26]. In accordance with the convention for numbering baculovirus ORFs [27], the ORF encoding granulin was designated as ORF1, and the adenine of the *granulin* ORF start codon was designated as nt 1 of the genome.

Homologous region (*hr*) sequences were identified using the pattern- and repeat-finding functions of the LaserGene GeneQuest program. Individual repeats from the *hr*s were aligned using Clustal W in LaserGene MegAlign (v. 12), and the alignment and repeat consensus sequence were displayed with BOXSHADE (http://www.ch.embnet.org/software/BOX_form.html).

### Sequence comparison and phylogeny

Shared synteny between MyunGV#8 and Spodoptera frugiperda granulovirus isolate VG008 (SpfrGV-VG008) [28], Xestia c-nigrum granulovirus isolate α4 (XecnGV- α4) [29], and Cydia pomonella granulovirus isolate M1 (CpGV-M1) [30] was determined by constructing gene parity plots as previously described [31].

Pairwise Kimura-2-parameter nucleotide distances between MyunGV#8 and other clade *a* betabaculoviruses were determined for partial *lef-8*, *lef-9*, and *polh* sequences as previously described [6] using MEGA6.

For phylogenetic inference, amino acid sequences were aligned by Clustal W using LaserGene MegAlign 12 (DNASTAR) with default parameters, except for the core gene amino acid sequences of desmoplakin (AC66) and AC78. For these alignments, the multiple and pairwise alignment penalties were reduced from 10 to 5 and the multiple alignment gap length penalty was reduced from 0.2 to 0.1 to compensate for the lower degree of conservation among the sequences of these proteins. For the core gene phylogeny, the amino acid alignments were concatenated using BioEdit 7.1.3 prior to analysis. The sequences used for phylogenetic inference are listed in S1 Table.

Phylogenetic trees were constructed using MEGA6 with the minimum evolution (ME) and maximum likelihood (ML) methods with 500 bootstrap replicates. The best-fitting evolutionary models and the value for the shape parameter for modeling rate differences among sites were determined from the alignments.

## Results and Discussion

### Ultrastructural observations on MyunGV#8 occlusion bodies

**Fig 1:**
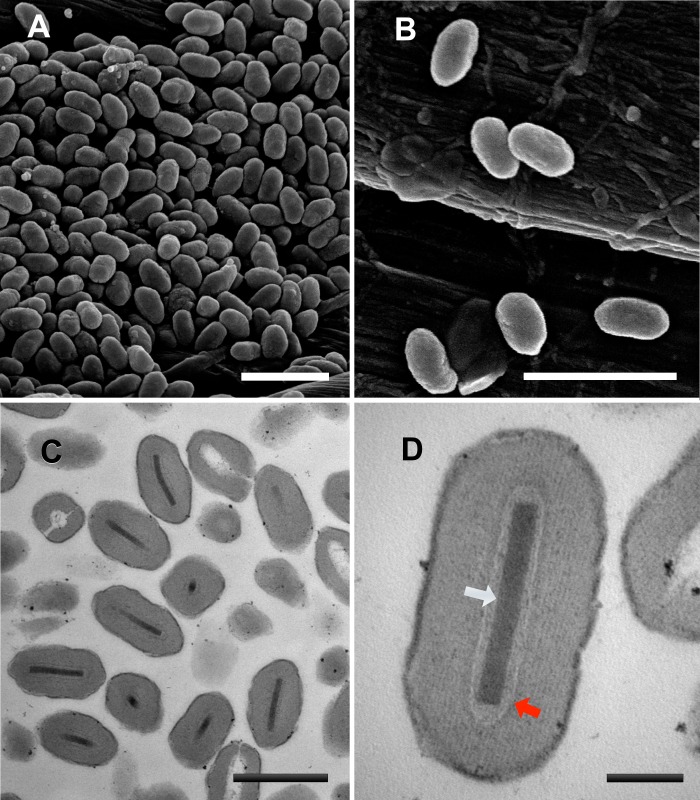
Occlusion bodies of betabaculovirus isolate MyunGV#8. (A, B) Scanning electron micrographs of MyunGV#8 occlusion bodies. (C, D) Transmission electron micrographs of ultrathin sections through MyunGV#8 occlusion bodies. The nucleocapsid (white arrow) and surrounding envelope (red arrow) of an occluded virion are indicated in panel D. Scale bars: 1 μm (A, B), 500 nm (C), 100 nm (D).

Occlusion bodies of the MyunGV#8 sample visualized by SEM and TEM exhibited the ovocylindrical shape characteristic of betabaculovirus occlusion bodies [1] (Fig 1A and Fig 1B). Each OB contained a single virion composed of a single enveloped nucleocapsid (Fig 1C and Fig 1D). From TEM cross sections, MyunGV#8 OBs spanned approximately 550 nm X 250 nm, which falls within the range of dimensions reported for betabaculovirus OBs [1]. The nucleocapsids also were of an expected length (approximately 250 nm), and no differences in the appearance of the ends of the nucleocapsids could be discerned.

### Confirmation of host species by barcoding PCR

The label for MyunGV#8 provided no species epithet for the host from which the virus was isolated, suggesting that there may have been some uncertainty on the part of the collector regarding the identity of the host. To confirm the host species for this virus isolate, barcoding PCR was carried out using COI primers on DNA isolated from an uninfected *M*. *unipuncta* larva and from the supernatant of a centrifuged aliquot of MyunGV#8. Both templates yielded an identical 439-bp amplimer with a sequence that matched 37 entries for *M*. *unipuncta* COI in GenBank with 99.8–100% nucleotide sequence identity. The next best match was with COI sequences from *Mythimna separata*, with 96.4% sequence identity. A parallel control reaction set up with DNA from Sf9 cells yielded an amplimer with 100% identity to COI sequences from *Spodoptera frugiperda*. These results confirm that the MyunGV#8 sample had been isolated from larvae of *M*. *unipuncta*, indicating that the true armyworm likely is the natural host for this virus isolate. Although the possibility that MyunGV#8 infects other moth species cannot be excluded, reports of betabaculovirus transmission to host species other than the species of origin are relatively rare [32]. *M*. *unipuncta* moths are commonly found in the U.S. Pacific Northwest that was thought to be the source of most of the Martignoni virus collection. Unfortunately, our efforts to establish a *M*. *unipuncta* colony to examine the host range and infectivity of MyunGV#8 failed due to the occurrence of alphabaculoviruses and other pathogens already present in the insects used to initiate the colony.

### Properties of the MyunGV#8 genome

**Fig 2:**
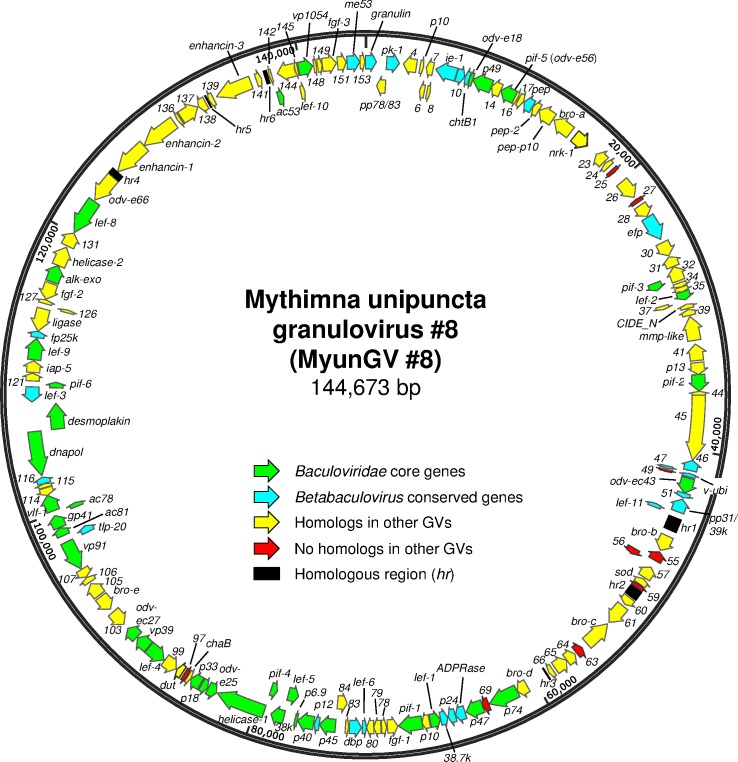
Map of the ORFs and other features of the MyunGV#8 genome. ORFs are represented by arrows, with the position and direction of the arrow indicating ORF position and orientation. Each ORF is designated by its number in the genome annotation (or, in the case of conserved or well-characterized baculovirus orthologues, a name). Categories of ORFs are indicated in the figure. Homologous repeat regions (*hr*s) are represented by black rectangles.

The final contig for the MyunGV#8 genome assembled from 454 and Sanger dideoxy sequencing data yielded a genome of 144,673 bp (Fig 2), significantly smaller than the 176,677-bp PsunGV-H genome. The sequence for MyunGV#8 possessed a nucleotide distribution of 49.9% G+C, which is considerably higher than the 39.8% G+C nucleotide distribution of the PsunGV-H genome sequence. The magnitude of the difference in nucleotide distribution between these two viruses may serve to reduce competition for nucleotide resources during virus replication in *M*. *unipuncta* larvae co-infected with MyunGV#8 and PsunGV-H [33]. A total of 153 ORFs were annotated in the MyunGV#8 genome, including 83 ORFs in the sense direction and 72 ORFs in the antisense direction (Fig 2, S2 Table).

**Fig 3:**
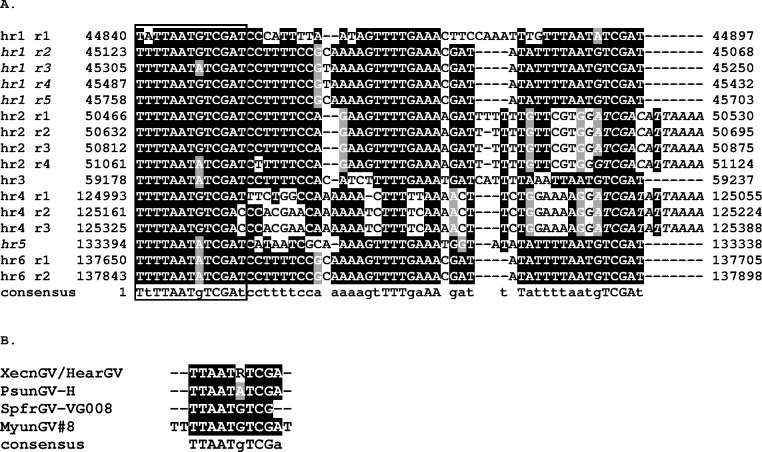
MyunGV#8 homologous region (*hr*) unit repeats. (A) An alignment of all unit repeats from MyunGV#8 *hr*s. Nucleotide positions of the repeats in the genome sequence are indicated. The conserved 13-bp terminal sequence is indicated by a box surrounding the aligned sequence at the 5’ terminus. Conserved terminal repeat sequences are present in a direct orientation except for the terminal sequences in *hr2* and *hr4*, which occur in an inverted orientation and are denoted in italics. Names of *hr* repeats occurring in an antisense orientation in the alignment are also indicated in italics. (B) Alignment of conserved terminal sequences from the *hr*s of other clade *a* betabaculoviruses. For both (A) and (B), identical nucleotides occupying >50% of aligned positions are shaded in black, and nucleotides of the same class as conserved nucleotides (containing either a purine or pyrimidine base) are shaded in gray. Nucleotides in the consensus sequence are denoted by uppercase letters for positions in the alignment with completely identical residues, and lowercase letters for positions in the alignment with a majority of identical residues.

Six homologous repeat regions (*hr*s) were also identified in the MyunGV#8 genome. The *hr*s consisted of 1–5 imperfect unit repeats ranging in size from 56 to 65 bp and bound by repeats with the consensus sequence TTTTAATGTCGAT (Fig 3A). In a manner previously reported for the XecnGV- α4 genome [29], the conserved terminal sequences for some of the *hr* unit repeat sequences were directly repeated, and for other unit repeats the terminal repeat sequences were inverted with respect to each other, with the *hr* unit sequence forming an imperfect palindrome. The terminal sequences shared high sequence identity (90% over 10 bp) with the conserved terminal repeats found in the *hr*s of XecnGV- α4, Helicoverpa armigera granulovirus (HearGV), SpfrGV-VG008, and PsunGV-H (Fig 3B). These virus isolates contain 8 (SpfrGV-VG008) or 9 (XecnGV- α4, HearGV, PsunGV-H) *hr*s. Six of these *hr*s are conserved in MyunGV#8 on the basis of positions relative to conserved ORFs [28]. The MyunGV#8 genome did not contain sequences corresponding to *hr1* and *hr5/5a* found in the genomes of the other virus isolates. The *hr*s likely function in mediating viral DNA replication, as concluded from the demonstrated activity as origins of DNA replication that *hr*s of CpGV and Cryptophebia leucotreta GV (CrleGV) exhibited in *C*. *pomonella* cell line-based replication assays [34, 35]. The *hr* elements in MyunGV#8 might also function as transcriptional enhancers as has been shown for some alphabaculoviruses [36, 37].

### Relationships to other baculoviruses

**Fig 4:**
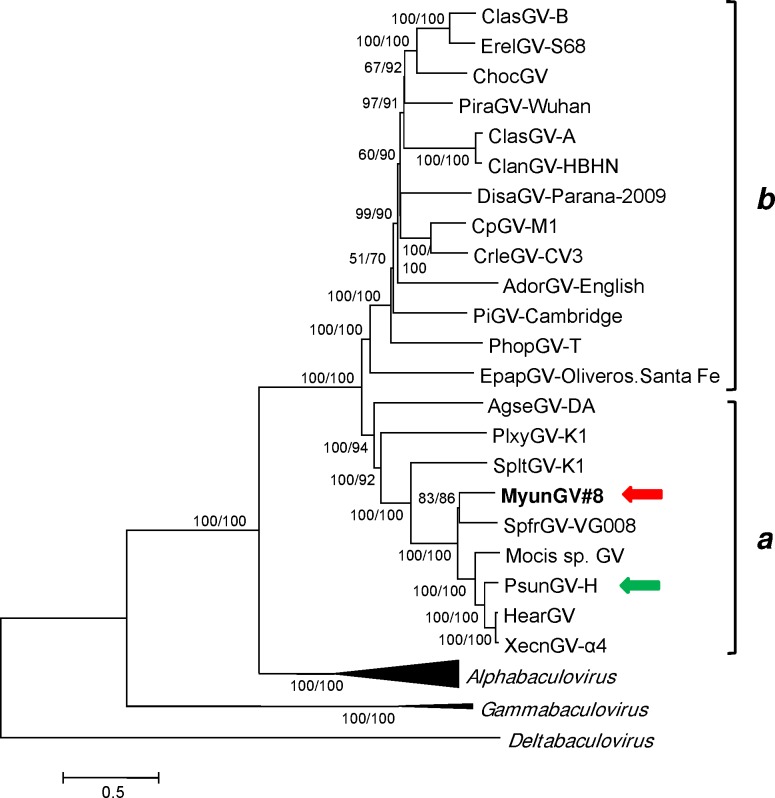
Relationships of MyunGV#8 and representative isolates of other baculovirus species, inferred from the predicted amino acid sequences of baculovirus core genes. The phylogenetic tree was constructed from the concatenated alignments of 37 baculovirus core gene amino acid sequences using the ME method. Branches for the genera *Alphabaculovirus*, *Deltabaculovirus*, and *Gammabaculovirus* are collapsed. The *a* and *b* clades of *Betabaculovirus* [38] are indicated with brackets. Bootstrap values >50% for both ME and ML analysis are indicated for each interior branch (ME/ML). In addition to MyunGV#8 (indicated by a red arrow) and PsunGV-H (indicated by a green arrow), virus taxa and sequences used in the analysis are as listed in S1 Table.

Phylogenetic inference of concatenated baculovirus core gene amino acid sequence alignments conducted with minimum evolution (ME) and maximum likelihood (ML) methods resulted in trees with the same topology and well-supported branches (Fig 4). The MyunGV#8 isolate was grouped with SpfrGV-VG008, while PsunGV-H was placed with XecnGV-α4 and HearGV. Both *M*. *unipuncta* betabaculovirus isolates were part of a larger group within betabaculovirus clade *a* [38] that also included Spodoptera litura granulovirus K1 (SpltGV-K1) and Mocis sp. granulovirus (GenBank accession no. KR011718).

Pairwise Kimura-2-parameter distances for partial *lef-8*, *lef-9*, and *granulin* nucleotide sequences indicated that MyunGV#8 represented a species distinct from other species represented by isolates in this group, including *Spodoptera frugiperda granulovirus* and *Pseudaletia unipuncta granulovirus*. Distances between MyunGV#8 and SpfrGV-VG008 (in substitutions/site) were 0.616 (*lef-8*), 0.503 (*lef-9*), and 0.2 (*granulin*). These values are well above the 0.050 demarcation limit proposed for separating two baculovirus species [6]. The nucleotide distances were larger for alignments between MyunGV#8 and other betabaculoviruses in clade *a*.

**Fig 5:**
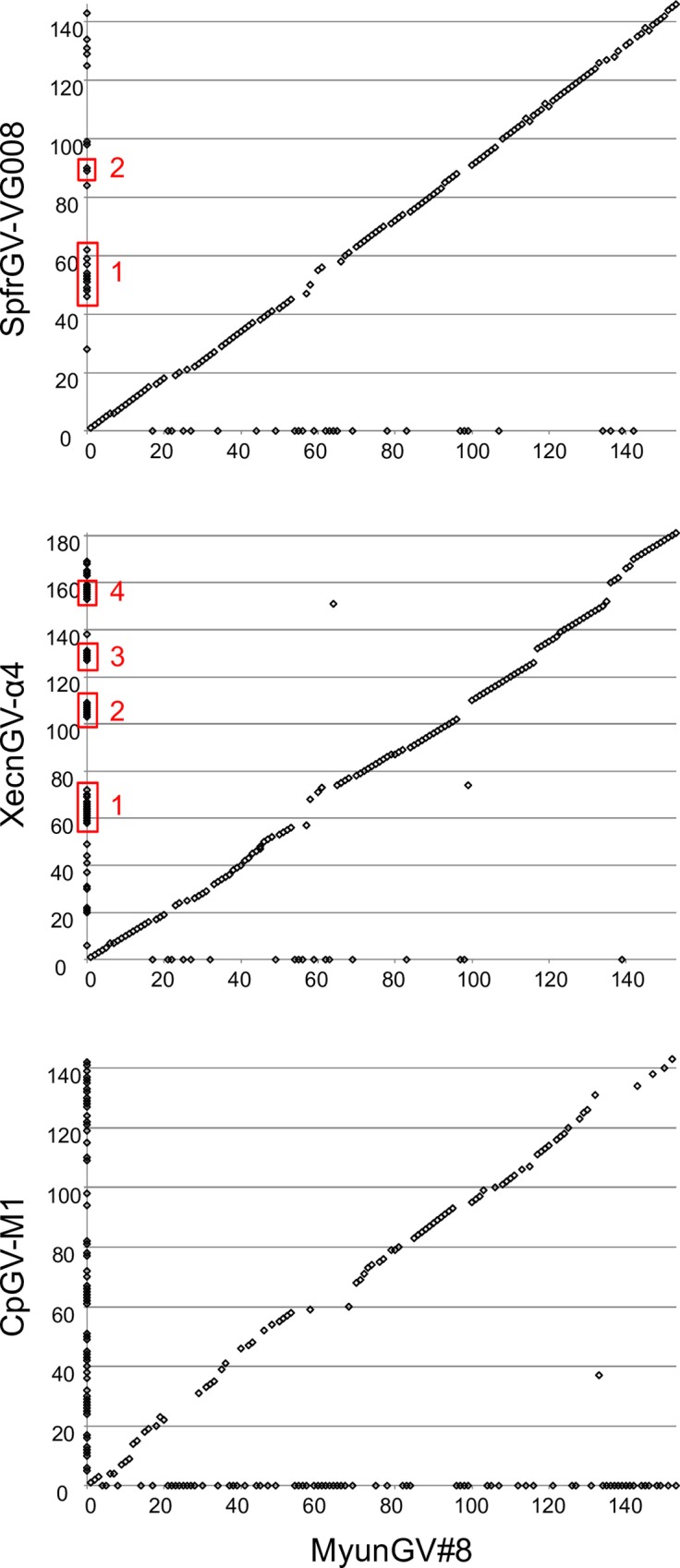
Gene parity plots comparing MyunGV#8 with representative clade *a* and *b* betabaculoviruses. Plots show the ORF content and order of the MyunGV#8 genome (x-axis) with that of SpfrGV-VG008, XecnGV-α4, and CpGV-M1 (y-axes). Each point in the plot represents an ORF. ORFs present in only one of the compared genomes appear on the axis corresponding to the virus in which they are present. Clusters of ORFs that are present in other clade *a* betabaculoviruses but not in MyunGV#8 are indicated by red rectangles numbered 1 through 4.

Gene parity plot analysis was carried out to visualize the synteny between the MyunGV#8 genome and the genomes of selected other betabaculoviruses (Fig 5). In general, the plots did not reveal any genomic inversions, even between MyunGV#8 and the clade *b* virus, CpGV-M1. The plot comparing MyunGV#8 with XecnGV-α4 revealed four clusters of ORFs in XecnGV-α4 that are missing from the MyunGV#8 genome (Fig 5, red boxes labeled 1 through 4). The absence of ORFs in MyunGV#8 corresponding to clusters 3 and 4 in XecnGV-α4 correlated with the complete absence of homologous nucleotide sequence in MyunGV#8 where those ORFs would be located. In contrast, the nucleotide sequence corresponding to the XecnGV-α4 ORFs in clusters 1 and 2 appears to have been at least partially replaced with a sequence containing a different set of ORFs in MyunGV#8. While homologues of the XecnGV-α4 ORFs in clusters 1–4 are also present in PsunGV-H, the ORFs in clusters 3 and 4 are also absent from SpfrGV-VG008 (S2 Table). The sites of genomic recombination in baculovirus genomes are often found near *hr*s [27], but only cluster 1 was located adjacent to an *hr* sequence.

### Gene content

In addition to the 37 core genes currently recognized for family *Baculoviridae* [39] (Fig 2, S2 Table), the MyunGV#8 genome also possesses homologs of most of the non-core genes found by Garavaglia et al. (2012) [39] to be conserved among all members of *Alphabaculovirus* and *Betabaculovirus*. Homologs for two of these conserved non-core genes, *gp37* and *exon0*, were not found in MyunGV#8. These genes also do not occur in the genomes of clade *a* GVs SpfrGV-VG008 and Agrotis segetum granulovirus (AgseGV-DA; GenBank accession no. KR584663), nor in the clade b GVs Erinnyis ello granulovirus S68 (ErelGV-S68) [40] and Plodia interpunctella granulovirus (PiGV-Cambridge) [41].

The MyunGV#8 genome contains five members of the *baculovirus repeated ORF* (*bro*) multigene family. Homologs of *bro* genes and genes bearing the Bro_N domain can be found in a broad cross-section of arthropod DNA viruses, bacterial phages, and bacteria [42, 43]. Among baculoviruses, the copy number of *bro* ORFs can range from 0 to 20. Sequences of *bro* genes within and among baculoviruses can be highly variable, suggesting that these sequences frequently undergo recombination. Of the five *bro* genes in MyunGV#8, only two (*bro-d* and *bro-e*) matched *bro* genes found in other clade *a* GVs. The other three *bro* genes (*bro-a*, *-b*, and–*c*) appear to be the results of duplication or insertion. In addition, MyunGV#8 contains three *enhancin* genes. In baculoviruses, these genes encode zinc metalloproteinases that have been shown in some cases to degrade mucin-like protein found in peritrophic matrix and augment the accessibility of midgut epithelial cells to infection by ODV from the occlusion bodies [44–46]. Enhancins were first identified as the “synergistic factor” found associated with PsunGV-H OBs that enhanced the pathogenicity of an *M*. *unipuncta* NPV in laboratory infections [47]. The PsunGV-H genome also contains three *enhancin* genes, while the XecnGV-α4 genome contains four such genes and the SpfrGV-VG008 genome contains two.

#### ORFs with no homologs in other betabaculoviruses

Nine ORFs were identified that did not contain homologs in other betabaculoviruses (Table 1). Five of these ORFs did not exhibit significant amino acid sequence similarity with other sequences or contain conserved domains or motifs when used in blastp or HHpred queries. In a blastp query with ORF56, the only two matches with an E-value <0.01 were ORFs 72 and 73 from Leucania separata nucleopolyhedrovirus AH1 (LeseNPV-AH1). Matches from a blastp query with the ORF59 amino acid sequence included several uncharacterized proteins from insect species. This ORF contained a copy of the chitin-binding domain type 2 (ChtBD2) motif. Both ORF56 and ORF59 were found by HHpred to possess significant sequence similarity to tachycitin, a small (73-amino acid) antimicrobial protein found in horseshoe crab (*Tachypleus tridentatus*) with a chitin-binding domain that is conserved among invertebrates and plants [48]). ORF56 encodes two direct repeats of the tachycitin sequence, while ORF59 encodes a single iteration. HHpred queries with the amino acid sequences of Autographa californica multiple nucleopolyhedrovirus C6 (AcMNPV-C6) ORFs *ac145* and *ac150* also yielded matches to tachycitin with 99+% true-positive probabilities. The predicted proteins of *ac145* and *ac150* play a role in the oral infectivity of AcMNPV, albeit in a host-specific fashion [49]. These results suggest that the putative ORF56 and ORF59 gene products may also influence oral infectivity of MyunGV#8.

**Table 1:** MyunGV#8 ORFs with no homologs in other betabaculovirus genomes.

ORF	Position/Size (aa)	Promoter motifs^a^	Best blastp match (E < 0.01)	Notes^b^
25	19091→19345/84	-	-	
27	21714←21968/84	L	-	
49	41659→41901/80	-	unknown [Zea mays], E = 1.6e^-12^	
55	47450←48100/216	-	-	
56	48090→48542/150	L	ORF73 [Leucania separata nucleopolyhedrovirus]; E = 2e^-23^	ORF72 [Leucania separata nucleopolyhedrovirus] E = 8e^-20^; Family g.31.1.1: Tachycitin, prob = 98.9% (N-terminal copy) and 99.1% (C-terminal copy),
59	50086←50373/95	-	uncharacterized protein Dwil_GK16219 [Drosophila willistoni]; E = 2e^-6^	Chitin-binding domain type 2 (ChtBD2); E = 1.21e^-7^; Family g.31.1.1: Tachycitin, prob = 99.4%
63	56152←56682/176	E	asb016 [Agrotis segetum nucleopolyhedrovirus B]; E = 3e^-28^	
69	63729→64154/141	-	-	
97	85384←85641/85	L	-	

^a^ TATAA (E) or TAAG (L) transcriptional promoter motifs present within 200 bp of the start codon.

^b^ E-values are from blastp queries; true-positive probabilities (prob) are from queries with HHpred.

MyunGV#8 ORF98 also encodes a homolog of deoxyuridine triphosphate nucleotidohydrolase (dUTPase; *dut*). Homologs of *dut* are found in both alpha- and betabaculoviruses, but the MyunGV#8 *dut* gene appears to belong to a distinct lineage. A blastp query with the MyunGV#8 DUT amino acid sequence yielded matches mostly to DUT sequences from insects and other animals, with one match to a DUT sequence from the clade *b* Epinotia aporema granulovirus (EpapGV). While the MyunGV#8 ORF is flanked by conserved homologs of AcMNPV-C6 ORFs *ac60* and *ac90*, the EpapGV-S68 *dut* is located upstream of its *envelope fusion protein* (*efp*) homolog, which corresponds to ORF29 in MyunGV#8. Likewise, SpfrGV-VG008 and SpltGV-K1 both contain *dut* genes, but they are located upstream of *superoxide dismutase* (*sod*), which corresponds to ORF58 in MyunGV#8. The blastp results and the discordant locations of the *dut* ORFs suggest that MyunGV#8 *dut* may not belong to the same lineage as the SpfrGV-VG008, SpltGV-K1, or EpapGV *dut* ORFs. Previous analysis of viral and host *dut* genes from a variety of sources supports the horizontal transfer of *dut* genes from host to virus genomes and the subsequent duplication, deletion and movement of *dut* genes within viral genomes [50]. Two comprehensive phylogenetic analyses of baculovirus *dut* genes have been published recently [51, 52]. Both analyses indicate that baculovirus *dut* genes do not constitute a monophyletic group, but likely originate from multiple sources. Ardisson-Araujo and coworkers (2016) [51] conclude from their analysis that baculovirus *dut* genes are the result of ten different acquisition events. It is possible that the MyunGV#8 *dut* gene was also a consequence of a separate horizontal gene transfer.

#### ORFs with closely related homologs in the LeseNPV-AH genome

**Fig 6:**
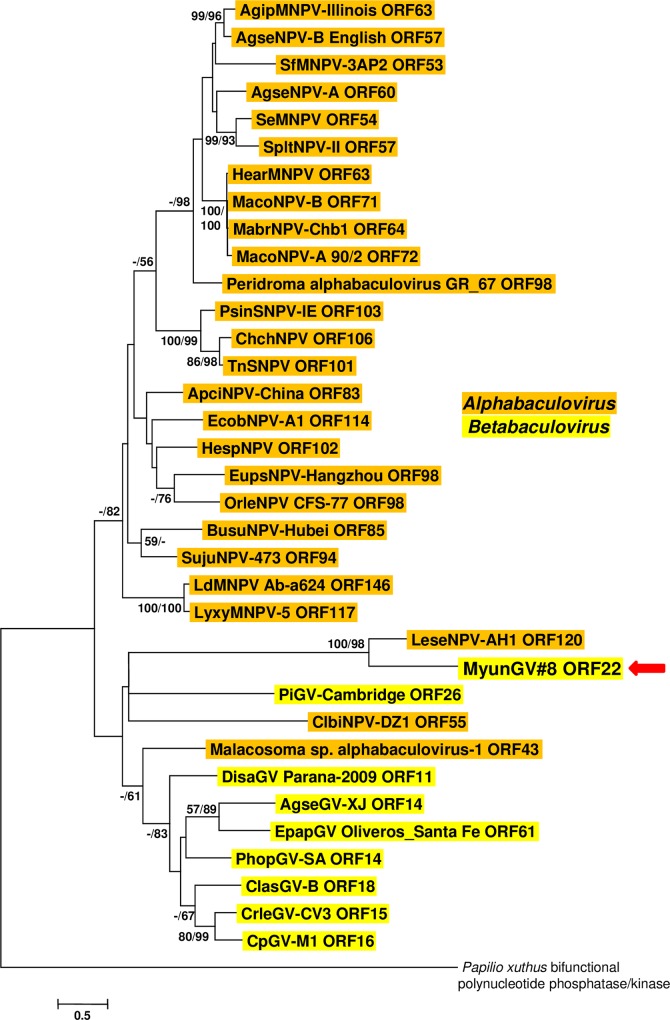
Phylogenetic analysis of baculovirus NRK (*nrk-1*) homologs. ML phylogram inferred from the alignment of *nrk-1*-encoded amino acid sequences is shown with bootstrap values (>50%) at interior branches for ME and ML analysis (ME/ML) where they occur. The sequence for *Papilio xuthus* bifunctional polynucleotide phosphatase/kinase (GenBank accession no. KPI94435) was included as an outgroup. In addition to MyunGV#8 (indicated by a red arrow), virus taxa are as indicated in S1 Table. The genus of each taxon is indicated with a color-coded text background.

In addition to ORF56, three other MyunGV#8 ORFs–ORF22, ORF41, and ORF136—exhibited significant sequence similarity with ORFs of LeseNPV-AH1. The highest-scoring blastp match for MyunGV#8 ORF22 was with LeseNPV-AH1 ORF120, with which it shared 49.2% amino acid sequence identity. ORF22 encodes a nicotinamide riboside kinase (NRK), which phosphorylates nicotinamide riboside to produce nicotinamide mononucleotide [53]. Several group II alphabaculoviruses contain homologs of this gene, abbreviated as *nrk-1*. Although some *nrk-1* ORFs also occur in the genomes of other betabaculoviruses, none of the betabaculovirus NRK1 sequences appeared in the results of a blastp query with ORF22. Alignment and phylogenetic inference of ORF22 and related sequences from other baculoviruses grouped the MyunGV#8 and LeseNPV-AH1 homologs together (Fig 6). Although alphabaculovirus and betabaculovirus-specific clusters of NRK1 homologs were evident from this analysis, the relationships of the MyunGV#8 and LeseNPV homologs to other baculovirus NRK1 homologs were unclear.

**Fig 7:**
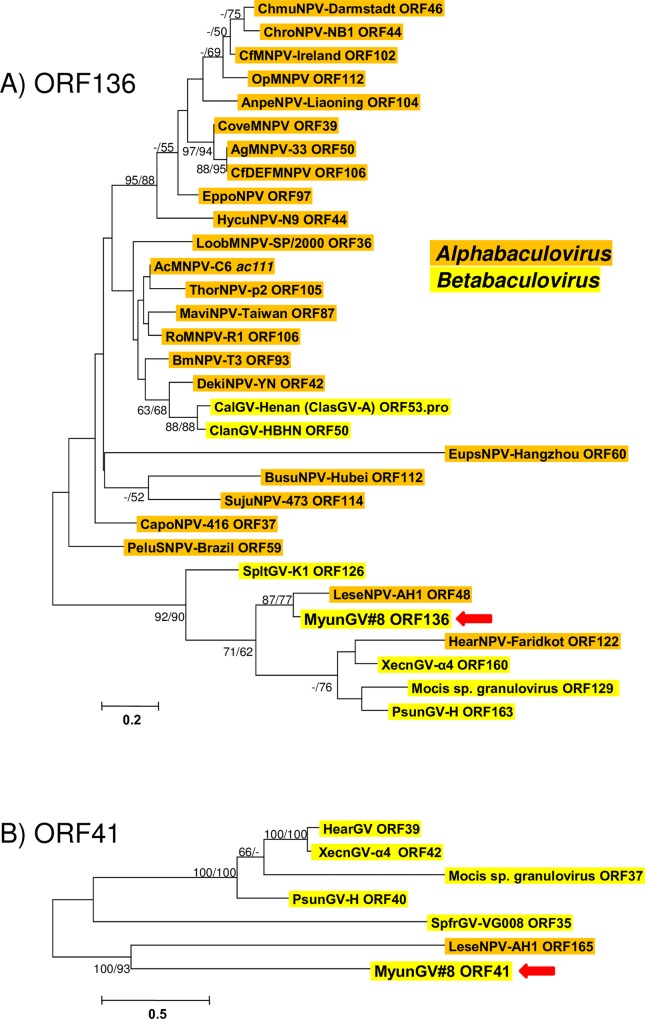
Phylogenetic analysis of MyunGV#8 ORF136 and ORF41 homologs. (A) ML phylogram inferred from the alignment of encoded amino acid sequences encoded by homologs of MyunGV#8 ORF136 (*ac111)*. (B) ORF41 ML phylogram inferred from alignment of ORF41 homolog amino acid sequences. In both (A) and (B), bootstrap values (>50%) are shown at interior branches for ME and ML analysis (ME/ML) where they occur. In addition to MyunGV#8 (indicated by a red arrow), virus taxa are as indicated in S1 Table. The genus of each taxon is indicated with a color-coded text background.

MyunGV#8 ORFs 41 and 136 also numbered LeseNPV-AH1 ORFs among their top matches in blastp queries, with amino acid sequence identities of 29.7% and 78.8%, respectively. Homologs for ORFs 41 and 136 were detected in the genomes of other clade *a* betabaculoviruses (S2 Table), and phylogenetic trees with these sequences suggest that ORFs 41 and 136 were present in an ancestral clade *a* betabaculovirus prior to its divergence into the current taxa (Fig 7). The LeseNPV-AH1 homologs for these ORFs group with the MyunGV#8 sequences with good bootstrap support, and the sums of branch lengths separating the MyunGV#8 and LeseNPV-AH1 sequences in both trees are less than the sums of the branch lengths separating the MyunGV#8 ORFs from their homologs in the other clade *a* viruses. These observations suggest that a recent ancestor of MyunGV#8 may have served as the source for these ORFs for a recent ancestor of LeseNPV-AH1.

ORF136 is a homolog of AcMNPV-C6 ORF *ac111*, a small (67-codon) ORF which is present in all group I alphabaculoviruses and a small number of group II alphabaculoviruses. It is also present in some clade *a* betabaculoviruses and two clade *b* betabaculoviruses (ClasGV-A and ClanGV-HBHN). The clade *b* ORF136 homologs appear to be derived from a group I alphabaculovirus lineage (Fig 7A). A homolog of this ORF was also detected in a number of isolates from Helicoverpa armigera nucleopolyhedrovirus (HearNPV). The HearNPV ORF with the best match to ORF136 was ORF122 of isolate HearNPV-Faridkot (GenBank accession number AIY24927), which is the homolog of ORF116 in *Helicoverpa armigera nucleopolyhedrovirus* reference isolate HearNPV-G4. This ORF grouped with the corresponding homologs from clade *a* betabaculoviruses, suggesting that the HearNPV lineage, like the LeseNPV-AH1 lineage, obtained its *ac111* homolog from a betabaculovirus.

Unlike MyunGV#8 ORF136, homologs of MyunGV#8 ORF41 only occur among betabaculoviruses of clade *a*, and phylogenetic inference grouped the LeseNPV-AH1 homolog of this ORF with the MyunGV#8 ORF41 (Fig 7B).

#### ORFs found in clade *a*, but not clade *b*, betabaculoviruses

During blastp searches with MyunGV#8 ORFs, we discovered that a set of 31 ORFs that occur only in betabaculoviruses of clade *a* (Table 2). Some of these ORFs have homologs in select alphabaculoviruses, ascoviruses, and entomopoxviruses, but not in betabaculoviruses of clade *b*. The actual distribution of individual ORFs in this set among clade *a* virus isolates varies from ORF to ORF, and an analysis of ORFs in other clade a viruses may reveal additional clade *a*-specific ORFs that are not present in the MyunGV#8 genome. The genomes of AgseGV-DA and PlxyGV-K1 only appear to contain six and four of these ORFs, respectively, that have been annotated in their genomes.

**Table 2:** ORFs specific to betabaculoviruses of clade *a*.

MyunGV#8	XecnGV/HearGV	PsunGV-H	Mocis sp. GV	SpfrGV-VG008	SpltGV-K1	PlxyGV-K1	AgseGV-DA	Notes^a^
ORF4	ORF4	ORF4	ORF4	ORF4	-	-	-	
ORF14	ORF14	ORF13	ORF13	ORF13	ORF12	ORF15	ORF14	Family g.44.1.2: U-box, prob = 99.2%
ORF17	-	-	-	-	ORF14	ORF18	-	Family g.40.1.1: Retrovirus zinc finger-like domains, prob = 99.0%
ORF23	ORF23	ORF22	-	ORF19	-	-	-	Family a.2.3.1: Chaperone J-domain, prob = 94.9%
ORF24	ORF24	ORF23	-	ORF20	-	-	-	Homologs also present in ascoviruses
ORF26	ORF25	ORF24	ORF23	ORF21	ORF22	-	ORF26	
ORF30	ORF28	-	ORF27	ORF24	-	-	-	
ORF34	ORF33	-	-	-	-	-	-	pfam05887: Trypan_PARP (Procyclic acidic repetitive protein), prob = 98.7%
ORF38	ORF38	ORF36	-	ORF32	ORF35	-	-	pfam02017: CIDE_N domain, E = 8.77e^-18^
ORF41	ORF42	ORF40	ORF37	ORF35	-	-	-	LeseNPV ORF165; E = 2e^-65^
ORF44	ORF46	ORF44	ORF42	-	ORF40	-	-	
ORF57	ORF57	ORF55	ORF51	ORF47	-	-	-	Homologs in *Mamestra* and *Spodoptera* spp. NPVs
ORF60	ORF71	ORF70	ORF55	ORF55	-	-	-	
ORF65	ORF74	ORF73	-	-	-	-	-	MyunGV#8 ORF99 is a duplicate of this ORF
ORF78	ORF86	ORF89	ORF71	-	-	-	-	
ORF84	ORF90	ORF94	ORF76	ORF76	ORF72	-	-	
ORF105	ORF115	ORF119	ORF97	ORF96	-	-	-	
ORF107	ORF117	ORF121	ORF99	-	-	-	-	
ORF114	ORF124	ORF128	ORF106	ORF107	ORF98	ORF90	ORF112	
ORF126	ORF142	ORF149	ORF119	ORF118	-	-	-	
ORF127	ORF143	ORF150	ORF120	ORF119	-	-	-	
ORF137	ORF161	ORF164	ORF130	ORF128	-	-	ORF139	Homologs in entomopoxviruses, ascoviruses, and MacoNPV-B; pfam13930 Endonuclea_NS_2 (DNA/RNA non-specific endonuclease), prob = 99.8%
ORF138	ORF162	ORF165	ORF131	ORF130	-	-	-	
ORF139	ORF164	ORF168	-	-	-	-	-	
ORF141	ORF167	ORF171	-	ORF133	-	-	-	
ORF142	ORF170	ORF172	ORF134	-	-	-	-	
ORF145	ORF173	ORF175	ORF137	ORF138	ORF129	-	ORF141	
ORF148	ORF176	ORF178	ORF139	ORF140	ORF131	ORF116	ORF144	
ORF149	ORF177	ORF179	ORF141	ORF141	-	-	-	
ORF151	ORF179	ORF181	ORF143	ORF143b	-	-	-	
ORF153	ORF181	ORF183	ORF145	ORF145	-	-	-	

^a^ E-values are from blastp queries; true-positive probabilities (prob) are from queries with HHpred.

One of the clade a-specific ORFs, MyunGV#8 ORF38, encodes a protein containing a CIDE_N domain. The CIDE_N domain is usually located at the N-terminus of the caspase-activated DNAse/DNA fragmentation factor (CAD/DFF) and CIDE (cell death-inducing DFF45-like effector) proteins, which are associated with breakdown of cellular chromosomal DNA during apoptosis [54]. CAD/DFF exists as a heterodimer consisting of DFF40/DFFB and DFF45/DFFA, which interact through the CIDE_N domains present in both proteins. DFFB possesses a double-stranded DNA-specific nuclease activity that is inhibited when bound to DFFA [55]. Cleavage of DFFA by caspase-3 releases active DFFB, which generates double-stranded breaks in chromatin leading to chromatin condensation and degradation. CIDE proteins also induce apoptosis and DNA fragmentation, though more recently they have been implicated in the regulation of lipid metabolism [56].

A blastp query with the ORF38 amino acid sequence returned a number of matches to insect sequences for DFFA (“DNA fragmentation factor subunit alpha-like”) proteins, as well as sequences labeled as CIDE proteins. Insects only appear to encode DFF proteins and not CIDE proteins [57], suggesting that the insect sequences identified as CIDE proteins may have been mislabeled.

**Fig 8:**
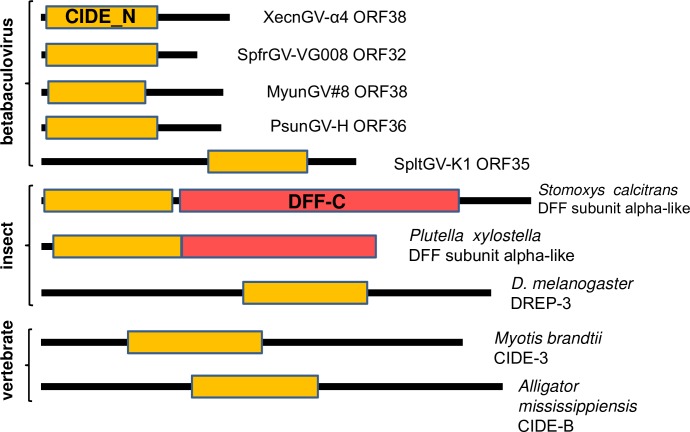
Structure of betabaculovirus, insect, and vertebrate CIDE_N domain-containing polypeptides. A schematic illustration of CIDE_N domain-containing polypeptides encoded by clade *a* betabaculoviruses and from selected insects and vertebrates with blastp matches. Lines representing the predicted polypeptides are drawn in proportion to their sizes. CIDE_N (orange rectangles) and DFF-C (red rectangles) domains are indicated. The size of the consensus CIDE_N domain is 58 amino acids.

CIDE_N-containing homologs of MyunGV#8 ORF38 were identified in XecnGV-α4, SpfrGV-VG008, PsunGV-H, and SpltGV-K1. The homologs of the first four betabaculoviruses specified predicted products ranging in size from 91 to 111 amino acids, while SpltGV-K1 ORF35 encoded a larger 185-amino acid product. The CIDE_N was positioned at the N-terminus in every predicted protein except that of SpltGV-K1, which occurred closer to the C-terminus (Fig 8). The insect DFFA homologs also contained CIDE_N domains positioned at the N-terminus, but encoded larger proteins (187–288 amino acids) that often contained a DFF-C domain [58]. This domain is found in the C-terminus of DFFA proteins and plays a role in proper folding of DFFB and inhibition of its DNAse activity.

**Fig 9:**
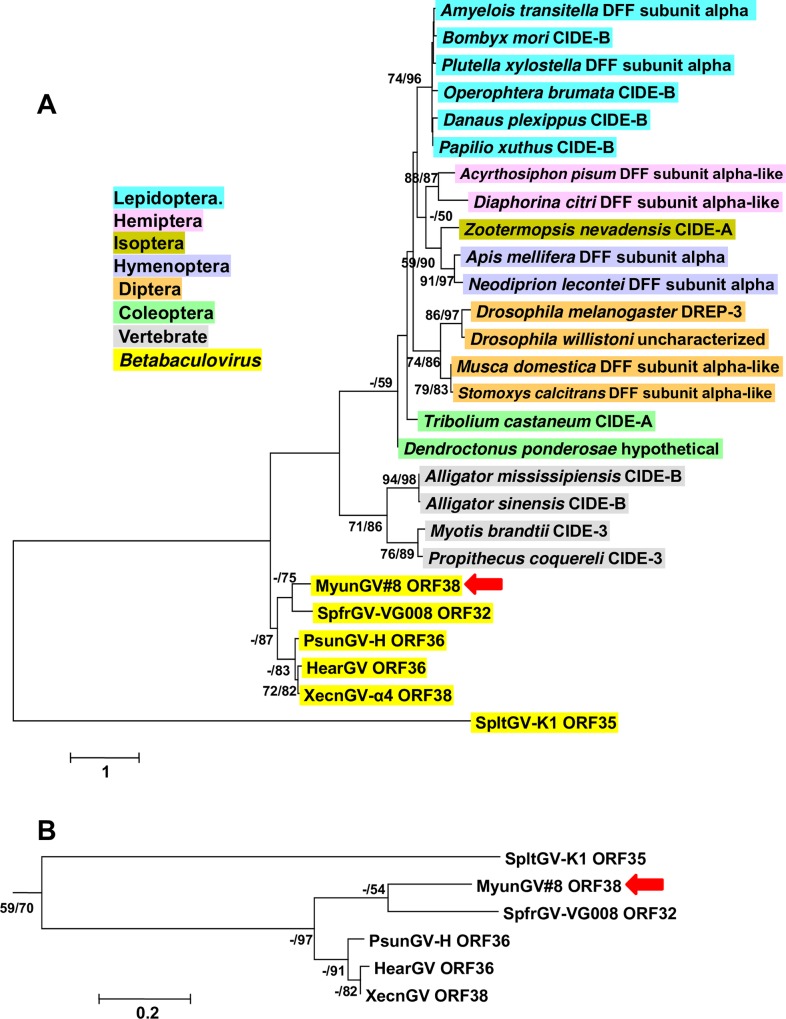
Phylogenetic analysis of viral and cellular CIDE_N domain-containing polypeptides. **(A)** ML phylogram inferred from the alignment of baculovirus, insect, and vertebrate amino acid sequences are shown with bootstrap values (>50%) at interior branches for ME and ML analysis (ME/ML) where they occur. The baculovirus genus or insect order for each taxon is indicated with color-coded text background; vertebrate taxa are indicated with a gray background. The virus and insect taxa and their accession numbers are as listed in S1 Table. (B) Betabaculovirus clade for a phylogeny inferred from an alignment exclusively of the CIDE_N domain sequences from the taxa in (A). The MyunGV#8 taxon is indicated in both trees by a red arrow.

Phylogenetic inference was carried out with the betabaculovirus CIDE_N-containing amino acid sequences along with a selection of insect DFF and vertebrate CIDE sequences that had occurred in blastp searches with MyunGV#8 ORF38. The insect, vertebrate, and betabaculovirus sequences each segregated into a separate clade (Fig 9A). The SpltGV-K1 sequence did not group with any clade, but when the analysis was repeated with just the CIDE_N motif sequences, the SpltGV-K1 taxon was placed in a clade with the other betabaculovirus sequences (Fig 9B). While the betabaculovirus CIDE_N ORFs appear to derive from a common ancestral gene, the origin of this gene was not evident from the phylogeny.

## Conclusions

The MyunGV#8 genome sequence reported here highlights how the analysis of baculovirus genomes continues to reveal details about the relationships, history and divergence of these viruses. The MyunGV#8 genome disclosed a pattern pointing to an exchange of genetic material between viruses of lineages represented by MyunGV#8 and LeseNPV-AH1, or their recent ancestors. The LeseNPV-AH1 virus was originally isolated from the Oriental armyworm, *Mythimna* (*Leucania*) *separata*. *M*. *separata* and *M*. *unipuncta* are closely related, although populations of these species do not share the same geographic range. Larvae of *M*. *unipuncta* and *M*. *separata*, or a common ancestor or related species, may have served as hosts to viruses of both lineages, with co-infection of larvae occurring to an extent that four ORFs were exchanged between isolates of these lineages. A similar exchange of genes between a distantly related alphabaculovirus and a betabaculovirus has been observed with Mamestra configurata nucleopolyhedrovirus-B (MacoNPV-B) and a XecnGV-related betabaculovirus [59].

Analysis also revealed a set of 31 ORFs exclusive to the betabaculoviruses of clade *a*. This ORF set delineates and defines a betabaculovirus lineage that includes MyunGV#8. Two of the viruses currently placed in clade *a*, AgseGV-DA and PlxyGV-K1, share a relatively low proportion of these ORFs (6 and 4, respectively; Table 2). Assuming that the annotations for these two genome sequences are accurate, this observation suggests that isolates of *Agrotis segetum granulovirus* and *Plutella xylostella granulovirus* may actually be part of a lineage that is different and distinct from betabaculovirus clade *a* (Table 2).

One of these clade *a*-specific ORFs, MyunGV#8 ORF38, encodes a CIDE_N motif-containing polypeptide. DFFA proteins vary in size; while vertebrate DFFA proteins are generally >300 amino acids [60], predicted insect DFFA-like proteins appear to range from approximately 180 to 280 amino acids (Fig 8). In contrast, the CIDE_N proteins encoded by the clade *a* betabaculoviruses are significantly smaller (91–111 amino acids, with the exception of the SpltGV-K1 homolog) and may be too small to be fully functional as DFFA proteins. It is conceivable that they may interact with cellular DFFA or DFFB proteins in order to prevent the apoptosis-associated degradation of viral DNA. CAD/DFF has been reported to induce apoptotic chromatin condensation [61] in a manner that visually resembles the nuclear margination of host chromatin that occurs during baculovirus infection [62]. One can speculate from this resemblance that the betabaculovirus CIDE_N-containing gene products also may interact with host DFF proteins to promote the condensation and margination of cellular chromatin. Such scenarios resemble the mechanism by which Orgyia pseudotsugata multiple nucleopolyhedrovirus (OpMNPV) inhibitor-of-apoptosis protein 3 (IAP-3) inhibits baculovirus replication-induced apoptosis in host cells. Rather than binding to and inhibiting activator caspases directly, OpMNPV IAP-3 binds and stabilizes host cell IAP, which in turn continues to prevent the onset of apoptosis [63].

It is expected that the continued sequencing and comparative analysis of novel baculovirus genomes will extend our comprehension of the evolution of baculoviruses and their genetic diversity.

## Supporting Information

S1 Table Names, abbreviations, and GenBank accession numbers of taxa used in phylogenetic inference. (DOCX)

S2 Table MyunGV#8 open reading frames (ORFs) and homologous repeat regions (*hr*s). (DOCX)
