# Supplementary material for: The Complete Genome Sequence of a Second Distinct Betabaculovirus from the True Armyworm, Mythimna unipuncta
Source: PLoS One. 2017 Jan 19;12(1):e0170510. doi: 10.1371/journal.pone.0170510 (PMC5245865; doi:10.1371/journal.pone.0170510)
Supplement: S1 Table — (DOCX) [file pone.0170510.s001.docx]

S1 Table. Names, abbreviations, and GenBank accession numbers of taxa used in phylogenetic inference.

| Virus isolate or insect species | Virus abbreviation | Order, family, and/or genus | Genbank accession numbers | | | | |
| --- | --- | --- | --- | --- | --- | --- | --- |
|  |  |  | Core genes (Fig 4)^a^ | NRK-1 homologs (Fig 6) | Ac111 homologs (Fig 7A) | MyunGV#8 ORF41 homologs (Fig 7B) | CIDE_N polypeptides (Fig 9) |
| Agrotis ipsilon multiple nucleopolyhedrovirus | AgipMNPV-Illinois | *Baculoviridae: Alphabaculovirus* | - | YP_002268093 | - | - | - |
| Agrotis segetum nucleopolyhedrovirus | AgseNPV-A | *Baculoviridae: Alphabaculovirus* | DQ123841 | YP_529730 | - | - | - |
| Agrotis segetum nucleopolyhedrovirus B | AgseNPV-B | *Baculoviridae: Alphabaculovirus* | - | YP_009112618 | - | - | - |
| Antheraea pernyi nucleopolyhedrovirus Liaoning | AnpeNPV-Liaoning | *Baculoviridae: Alphabaculovirus* | - | - | YP_611015 | - | - |
| Anticarsia gemmatalis multiple nucleopolyhedrovirus 2D | AgMNPV-2D | *Baculoviridae: Alphabaculovirus* | DQ813662 | - | - | - | - |
| Anticarsia gemmatalis multiple nucleopolyhedrovirus 33 | AgMNPV-33 | *Baculoviridae: Alphabaculovirus* | - | - | ALR70957 | - | - |
| Apocheima cinerarium nucleopolyhedrovirus-China | ApciNPV-China | *Baculoviridae: Alphabaculovirus* | - | YP_006607852 | - | - | - |
| Autographa californica multiple nucleopolyhedrovirus C6 | AcMNPV-C6 | *Baculoviridae: Alphabaculovirus* | L22858 | - | NP_054141 | - | - |
| Bombyx mori nucleopolyhedrovirus T3 | BmNPV-T3 | *Baculoviridae: Alphabaculovirus* | L33180 | - | NP_047511 | - | - |
| Buzura suppressaria nucleopolyhedrovirus | BusuNPV | *Baculoviridae: Alphabaculovirus* | - | YP_009001862 | YP_009001889 | - | - |
| Catopsilia pomona nucleopolyhedrovirus 416 | CapoNPV-416 | *Baculoviridae: Alphabaculovirus* | - | - | YP_009255294 | - | - |
| Choristoneura fumiferana multiple nucleopolyhedrovirus | CfMNPV | *Baculoviridae: Alphabaculovirus* | - | - | NP_848413 | - | - |
| Choristoneura fumiferana DEF multiple nucleopolyhedrovirus | CfDEFMNPV | *Baculoviridae: Alphabaculovirus* | - | - | NP_932715 | - | - |
| Choristoneura murinana nucleopolyhedrovirus Darmstadt | ChmuNPV-Darmstadt | *Baculoviridae: Alphabaculovirus* | - | - | YP_008992138 | - | - |
| Choristoneura rosaceana nucleopolyhedrovirus NB1 | ChroNPV-NB1 | *Baculoviridae: Alphabaculovirus* | - | - | YP_008378397 | - | - |
| Chrysodeixis chalcites nucleopolyhedrovirus | ChchNPV | *Baculoviridae: Alphabaculovirus* | - | AGE61512 | - | - | - |
| Clanis bilineata nucleopolyhedrovirus-DZ1 | ClbiNPV-DZ1 | *Baculoviridae: Alphabaculovirus* | - | YP_717593 | - | - | - |
| Condylorrhiza vestigialis multiple nuclepolyhedrovirus Brazil | CoveMNPV-Brazil | *Baculoviridae: Alphabaculovirus* | - | - | YP_009118522 | - | - |
| Dendrolimus kikuchii nucleopolyhedrovirus YN | DekiNPV-YN | *Baculoviridae: Alphabaculovirus* | - | - | AFS51920 | - | - |
| Ectropis obliqua nucleopolyhedrovirus | EcobNPV-A1 | *Baculoviridae: Alphabaculovirus* | - | YP_874307 | - | - | - |
| Epiphyas postvittana nucleopolyhedrovirus | EppoNPV | *Baculoviridae: Alphabaculovirus* | - | - | NP_203266 | - | - |
| Euproctis pseudoconspersa nucleopolyhedrovirus Hangzhou | EupsNPV-Hangzhou | *Baculoviridae: Alphabaculovirus* | - | YP_002854708 | YP_002854670 | - | - |
| Helicoverpa armigera multiple nucleopolyhedrovirus | HearMNPV | *Baculoviridae: Alphabaculovirus* | - | ACH88585 | - | - | - |
| Helicoverpa armigera nucleopolyhedrovirus G4 | HearNPV-G4 | *Baculoviridae: Alphabaculovirus* | AF271059 | - | - | - | - |
| Helicoverpa armigera nucleopolyhedrovirus Faridkot | HearNPV-Faridkot | *Baculoviridae: Alphabaculovirus* | - | - | AIY24927 | - | - |
| Hemileuca sp. nucleopolyhedrovirus | HespNPV | *Baculoviridae: Alphabaculovirus* | - | - | YP_008378318 | - | - |
| Hyphantria cunea nucleopolyhedrovirus | HycuNPV | *Baculoviridae: Alphabaculovirus* | - | - | YP_473232 | - | - |
| Leucania separata nucleopolyhedrovirus AH1 | LeseNPV-AH1 | *Baculoviridae: Alphabaculovirus* | - | YP_758417 | YP_758345 | YP_758462 | - |
| Lonomia obliqua multiple nucleopolyhedrovirus SP/2000 | LoobMNPV-SP/2000 | *Baculoviridae: Alphabaculovirus* | - | - | AKN81035 | - | - |
| Lymantria dispar multiple nucleopolyhedrovirus 5-6 | LdMNPV 5-6 | *Baculoviridae: Alphabaculovirus* | AF081810 | AMO27993 | - | - | - |
| Lymantria xylina multiple nucleopolyhedrovirus 5 | LyxyMNPV-5 | *Baculoviridae: Alphabaculovirus* | - | YP_003517857 | - | - | - |
| Malacosoma sp. alphabaculovirus 1 | - | *Baculoviridae: Alphabaculovirus* | - | ANW12276 | - | - | - |
| Mamestra brassicae nucleopolyhedrovirus K1 | MabrNPV-K1 | *Baculoviridae: Alphabaculovirus* | - | AFP95783 | - | - | - |
| Mamestra configurata nucleopolyhedrovirus A 90/2 | MacoNPV-A 90/2 | *Baculoviridae: Alphabaculovirus* | U59461 | NP_613155 | - | - | - |
| Mamestra configurata nucleopolyhedrovirus B | MacoNPV-B | *Baculoviridae: Alphabaculovirus* | - | NP_689246 | - | - | - |
| Maruca vitrata nucleopolyhedrovirus | MaviNPV | *Baculoviridae: Alphabaculovirus* | - | - | YP_950817 | - | - |
| Orgyia leucostigma nucleopolyhedrovirus CFS-77 | OrleNPV CFS-77 | *Baculoviridae: Alphabaculovirus* | - | YP_001651008 | - | - | - |
| Orgyia pseudotsugata multiple nucleopolyhedrovirus | OpMNPV | *Baculoviridae: Alphabaculovirus* | U75930 | - | NP_046268 | - | - |
| Peridroma alphabaculovirus GR_67 | - | *Baculoviridae: Alphabaculovirus* | - | YP_009049924 | - | - | - |
| Perigonia lusca single nucleopolyhedrovirus Brazil | PeluSNPV-Brazil | *Baculoviridae: Alphabaculovirus* | - | - | YP_009165659 | - | - |
| Rachiplusia ou multiple nucleopolyhedrovirus R1 | RoMNPV-R1 | *Baculoviridae: Alphabaculovirus* | - | - | AAN28146 | - | - |
| Spodoptera exigua multiple nucleopolyhedrovirus US | SeMNPV-US | *Baculoviridae: Alphabaculovirus* | AF169823 | NP_037814 | - | - | - |
| Spodoptera frugiperda multiple nucleopolyhedrovirus 3AP2 | SfMNPV-3AP2 | *Baculoviridae: Alphabaculovirus* | - | YP_001036345 | - | - | - |
| Spodoptera litura nucleopolyhedrovirus G2 | SpltNPV-G2 | *Baculoviridae: Alphabaculovirus* | AF325155 | - | - | - | - |
| Spodoptera litura nucleopolyhedrovirus II | SpltNPV-II | *Baculoviridae: Alphabaculovirus* | - | YP_002332755 | - | - | - |
| Sucra jujuba nucleopolyhedrovirus 473 | SujuNPV-473 | *Baculoviridae: Alphabaculovirus* | - | YP_009186785 | YP_009186805 | - | - |
| Thysanoplusia orichalcea nucleopolyhedrovirus P2 | ThorNPV-P2 | *Baculoviridae: Alphabaculovirus* | - | - | YP_007250517 | - | - |
| Trichoplusia ni single nucleopolyhedrovirus | TnSNPV | *Baculoviridae: Alphabaculovirus* | DQ017380 | YP_308990 | - | - | - |
| Adoxophyes orana granulovirus English | AdorGV-English | *Baculoviridae: Betabaculovirus* | AF547984 | - | - | - | - |
| Agrotis segetum granulovirus DA | AgseGV-DA | *Baculoviridae: Betabaculovirus* | KR584663 | - | - | - | - |
| Agrotis segetum granulovirus XJ | AgseGV-XJ | *Baculoviridae: Betabaculovirus* | - | YP_006330 | - | - | - |
| Choristoneura occidentalis granulovirus | ChocGV | *Baculoviridae: Betabaculovirus* | DQ333351 | - | - | - | - |
| Clostera anachoreta granulovirus HBHN | ClanGV-HBHN | *Baculoviridae: Betabaculovirus* | HQ116624 | - | YP_004376258 | - | - |
| Clostera anastomosis granulovirus A | CalGV-Henan, or ClasGV-A | *Baculoviridae: Betabaculovirus* | KC179784 | - | YP_008720000 | - | - |
| Clostera anastomosis granulovirus B | ClasGV-B | *Baculoviridae: Betabaculovirus* | KR091910 | AKS25361 | - | - | - |
| Cryptophlebia leucotreta granulovirus CV3 | CrleGV-CV3 | *Baculoviridae: Betabaculovirus* | AY229987 | NP_891862 | - | - | - |
| Cydia pomonella granulovirus M1 | CpGV-M1 | *Baculoviridae: Betabaculovirus* | U53466 | - | - | - | - |
| Cydia pomonella granulovirus I07 | CpGV-I07 | *Baculoviridae: Betabaculovirus* | - | AIU36805 | - | - | - |
| Diatraea saccharalis granulovirus Parana-2009 | DisaGV-Parana-2009 | *Baculoviridae: Betabaculovirus* | KP296186 | YP_009182209 | - | - | - |
| Epinotia aporema granulovirus Oliveros.Santa Fe | EpapGV-Oliveros.Santa Fe | *Baculoviridae: Betabaculovirus* | JN408834 | YP_006908569 | - | - | - |
| Erinnyis ello granulovirus S68 | ErelGV-S68 | *Baculoviridae: Betabaculovirus* | KJ406702 | - | - | - | - |
| Helicoverpa armigera granulovirus | HearGV | *Baculoviridae: Betabaculovirus* | EU255577 | - | - | YP_001649021 | ABY47727 |
| Mocis sp. granulovirus | - | *Baculoviridae: Betabaculovirus* | KR011718 | - | YP_009249966 | YP_009249874 | - |
| Phthorimaea operculella granulovirus T | PhopGV-T | *Baculoviridae: Betabaculovirus* | AF499596 | - | - | - | - |
| Phthorimaea operculella granulovirus SA | PhopGV-SA | *Baculoviridae: Betabaculovirus* | - | ANY57403 | - | - | - |
| Pieris rapae granulovirus Wuhan | PiraGV-Wuhan | *Baculoviridae: Betabaculovirus* | GQ884143 | - | - | - | - |
| Plodia interpunctella granulovirus Cambridge | PiGV-Cambridge | *Baculoviridae: Betabaculovirus* | KX151395 | - | - | - | - |
| Plutella xylostella granulovirus K1 | PlxyGV-K1 | *Baculoviridae: Betabaculovirus* | AF270937 | - | - | - | - |
| Pseudaletia unipuncta granulovirus H | PsunGV-H | *Baculoviridae: Betabaculovirus* | EU678671 | - | YP_003422502 | YP_003422379 | ACH69386 |
| Spodoptera frugiperda granulovirus VG008 | SpfrGV-VG008 | *Baculoviridae: Betabaculovirus* | KM371112 | - | - | YP_009121820 | AJK91693 |
| Spodoptera litura granulovirus K1 | SpltGV-K1 | *Baculoviridae: Betabaculovirus* | DQ288858 | - | YP_001257077 | - | ABQ51978 |
| Xestia c-nigrum granulovirus | XecnGV | *Baculoviridae: Betabaculovirus* | AF162221 | - | NP_059308 | NP_059190 | AF162221_38 |
| Neodiprion lecontei nucleopolyhedrovirus | NeleNPV | *Baculoviridae: Gammabaculovirus* | AY349019 | - | - | - | - |
| Neodiprion sertifer nucleopolyhedrovirus | NeseNPV | *Baculoviridae: Gammabaculovirus* | AY430810 | - | - | - | - |
| Culex nigripalpus nucleopolyhedrovirus Florida 1997 | CuniNPV-Florida 1997 | *Baculoviridae: Deltabaculovirus* | AF403738 | - | - | - | - |
| *Dendroctonus ponderosae* | - | Coleoptera: Curculionidae | - | - | - | - | ERL94534 |
| *Tribolium castaneum* | - | Coleoptera: Tenebrionidae | - | - | - | - | EEZ98068 |
| *Drosophila melanogaster* | - | Diptera: Drosophilidae | - | - | - | - | AF149796_1 |
| *Drosophila willistoni* | - | Diptera: Drosophilidae | - | - | - | - | EDW74559 |
| *Musca domestica* | - | Diptera: Muscidae | - | - | - | - | XP_005179615 |
| *Stomoxys calcitrans* | - | Diptera:Muscidae | - | - | - | - | XP_013106172 |
| *Acrythosiphon pisum* | - | Hemiptera: Aphididae | - | - | - | - | XP_001947580 |
| *Diaphorina citri* | - | Hemiptera: Psyllidae | - | - | - | - | XP_008467808 |
| *Apis mellifera* | - | Hymenoptera: Apidae | - | - | - | - | XP_001120965 |
| *Neodiprion lecontei* | - | Hymenoptera: Diprionidae | - | - | - | - | XP_015511236 |
| *Zootermopsis nevadensis* | - | Isoptera: Termopsidae | - | - | - | - | KDR23385 |
| *Amyelois transitella* | - | Lepidoptera: Pyralidae | - | - | - | - | XP_013191186 |
| *Bombyx mori* | - | Lepidoptera: Bombycidae | - | - | - | - | NP_001108342 |
| *Danaus plexippus* | - | Lepidoptera: Nymphalidae | - | - | - | - | EHJ77878 |
| *Operophtera brumata* | - | Lepidoptera: Geometridae | - | - | - | - | KOB64719 |
| *Papilio xuthus* | - | Lepidoptera: Papilionidae | - | KPI94435 | - | - | KPI94154 |
| *Plutella xylostella* |  | Lepidoptera: Plutellidae | - | - | - | - | XP_011555920 |
| *Alligator mississippiensis* | - | Crocodilia: Alligatoridae | - | - | - | - | XP_006259793 |
| *Alligator sinensis* | - | Crocodilia: Alligatoridae | - | - | - | - | XP_006039041 |
| *Myotis brandtii* | - | Chiroptera: Vespertilionidae | - | - | - | - | XP_005870146 |
| *Propithecus coquereli* | - | Primates: Indriidae | - | - | - | - | XP_012502312 |

^a^Accession numbers for genome sequences are given. Conceptual translations of core gene sequences from these genomes were used for the Fig 4 phylogeny.
