# Supplementary material for: The Complete Genome Sequence of a Second Distinct Betabaculovirus from the True Armyworm, Mythimna unipuncta
Source: PLoS One. 2017 Jan 19;12(1):e0170510. doi: 10.1371/journal.pone.0170510 (PMC5245865; doi:10.1371/journal.pone.0170510)
Supplement: S2 Table — (DOCX) [file pone.0170510.s002.docx]

S2 Table. MyunGV#8 open reading frames (ORFs) and homologous repeat regions (*hr*s)

| **ORF** | **Name** | **Position** | **Size (aa)** | **Orthologues in other betabaculoviruses (ORF#/% identity)^a^** | | | | **Notes/AcMNPV orthologues^c^** |
| --- | --- | --- | --- | --- | --- | --- | --- | --- |
|  |  |  |  | **PsunGV-H** | **SpfrGV-VG008^b^** | **XecnGV-α4** | **CpGV-M1** |  |
| **1** | *granulin* | 1🡪747 | 248 | 1 (98%) | 1 (95%) | 1 (98%) | 1 (88%) | *ac8* |
| **2** | *orf1629* capsid protein | 828🡨1478 | 216 | 2 (41%) | 3 (51%) | 2 (44%) | 2 (48%) | *ac9* |
| **3** | *pk-1* | 1459🡪2343 | 294 | 3 (69%) | 2 (79%) | 3 (69%) | 3 (43%) | *ac10* |
| **4** |  | 2615🡨3523 | 302 | 4 (31%) | 4 (35%) | 4 (28%) | - |  |
| **5** | *p10* | 3778🡪4026 | 82 | 5 (65%) | 5 (75%) | 5 (65%) | - | *ac137* |
| **6** |  | 4032🡨4391 | 119 | 6 (73%) | 7 (65%) | 7 (74%) | 4 (NSS) | ORF6 and ORF7 appear to occur as a single ORF in other viruses |
| **7** |  | 4208🡨4690 | 160 | 6 (50%) | 7 (53%) | 7 (53%) | 4 (44%) |  |
| **8** |  | 4587🡪4841 | 84 | 7 (54%) | 6 (67%) | 8 (58%) | - |  |
| **9** | *ie-1* | 4882🡨6336 | 484 | 8 (58%) | 8 (53%) | 9 (57%) | 7 (31%) | *ac147* |
| **10** |  | 6363🡪6944 | 193 | 9 (58%) | 9 (61%) | 10 (57%) | 8 (34%) | *ac146* |
| **11** | *chtb1* | 6965🡨7267 | 100 | 10 (83%) | 10 (76%) | 11 (83%) | 9 (51%) | *ac145* |
| **12** | *odv-e18* | 7277🡨7531 | 84 | 11 (85%) | 11 (81%) | 12 (86%) | 14 (60%) | *ac143* |
| **13** | *p49* | 7535🡨8896 | 453 | 12 (69%) | 12 (79%) | 13 (67%) | 15 (46%) | *ac142* |
| **14** |  | 8918🡨9592 | 224 | 13 (60%) | 13 (59%) | 14 (58%) | - | Family g.44.1.2: U-box, prob=99.2% |
| **15** | *pif-5* (*odv-e56*) | 9614🡨10672 | 352 | 14 (73%) | 14 (67%) | 15 (73%) | 18 (56%) | *ac148* |
| **16** |  | 10683🡪10895 | 70 | 15 (58%) | 15 (69%) | 16 (57%) | 19 (42%) | *ac29* |
| **17** |  | 10971🡪11354 | 127 | - | - | - | - | SlGV-K1 ORF14 E= 5e^-5^; Family g.40.1.1: Retrovirus zinc finger-like domains, prob=99.0% |
| **18** | *pep* | 11407🡨12063 | 218 | 16 (61%) | 16 (60%) | 17 (59%) | 20 (37%) | *ac131* |
| **19** | *pep-2* | 12098🡨12562 | 154 | 17 (84%) | 17 (86%) | 18 (83%) | 23 (51%) |  |
| **20** | *pep/p10* | 12582🡨13724 | 380 | 18 (80%) | 18 (77%) | 19 (79%) | 22 (51%) |  |
| **21** | *bro-a* | 13822🡨15240 | 472 | - | - | - | - | PsunGV-H ORF158, E= 0 |
| **22** | *nrk-1* | 15651🡪16727 | 358 | - | - | - | - | LeseNPV ORF120, E = 9e^-114^ |
| **23** |  | 17563🡨18327 | 254 | 22 (55%) | 19 (29%) | 23 (55%) | - | Family a.2.3.1: Chaperone J-domain, prob=94.9% |
| **24** |  | 18427🡨18795 | 122 | 23 (59%) | 20 (56%) | 24 (61%) | - | Ascovirus homologs |
| **25** |  | 19091🡪19345 | 84 | - | - | - | - |  |
| **26** |  | 20160🡪21533 | 457 | 24 (41%) | 21 (40%) | 25 (38%) | - |  |
| **27** |  | 21714🡨21968 | 84 | - | - | - | - |  |
| **28** |  | 22222🡪23112 | 296 | 25 (39%) | 22 (40%) | 26 (42%) | - |  |
| **29** | F protein (*efp*) | 23189🡪24964 | 591 | 26 (52%) | 23 (56%) | 27 (54%) | 31 (32%) | *ac23* |
| **30** |  | 25216🡪26220 | 334 | - | 24 (41%) | 28 (24%) | - |  |
| **31** |  | 26303🡨27040 | 245 | 28 (55%) | 25 (57%) | 29 (60%) | 33 (27%) |  |
| **32** |  | 27052🡨28092 | 346 | 29 (72%) | 26 (82%) | - | 34 (48%) |  |
| **33** | *pif-3* | 27647🡪28228 | 183 | 30 (72%) | 27 (66%) | 32 (71%) | 35 (42%) | *ac115* |
| **34** |  | 28243🡪28557 | 104 | - | - | 33 (67%) | - | pfam05887: Trypan_PARP (Procyclic acidic repetitive protein ), prob=98.7% |
| **35** |  | 28561🡪28893 | 110 | 32 (85%) | 29 (85%) | 34 (85%) | 39 (47%) |  |
| **36** | *lef-2* | 28897🡪29475 | 192 | 33 (57%) | 30 (59%) | 35 (59%) | 41 (37%) | *ac6* |
| **37** |  | 29459🡪29716 | 85 | 34 (43%) | 31 (48%) | 36 (43%) | - |  |
| **38** |  | 29790🡨30110 | 106 | 36 (60%) | 32 (70%) | 38 (64%) | - | pfam02017: CIDE_N domain, E=8.77e^-18^ |
| **39** |  | 30189🡨30602 | 137 | 37 (45%) | 33 (58%) | 39 (43%) | - |  |
| **40** | *mmp-like* | 30670🡨32364 | 564 | 38 (53%) | 34 (47%) | 40 (56%) | 46 (33%) |  |
| **41** |  | 32616🡨33767 | 383 | 40 (25%) | 35 (26%) | 42 (27%) | - | *ac111*; top match: LeseNPV ORF165, E = 2e-32 |
| **42** | *p13* | 33911🡪34735 | 274 | 41 (76%) | 36 (74%) | 43 (65%) | 47 (51%) |  |
| **43** | *pif-2* | 34742🡪35881 | 379 | 43 (82%) | 37 (75%) | 45 (80%) | 48 (53%) | *ac22* |
| **44** |  | 35894🡨36148 | 84 | 44 (38%) | - | 46 (40%) | - |  |
| **45** |  | 36167🡪40690 | 1507 | 45 (43%) | 38 (51%) | 47 (65%); 48 (32%) | 50 (NSS) | pfam09787: Golgin_A5 (Golgin subfamily A member 5), prob=99.9% |
| **46** |  | 40703🡨41416 | 237 | 47 (68%) | 39 (76%) | 50 (79%) | 52 (65%) | *ac106/107* |
| **47** |  | 41467🡪41646 | 59 | 48 (75%) | 40 (67%) | 51 (79%) | 53 (43%) | *ac110* |
| **48** | *v-ubi* | 41620🡨41862 | 80 | 50 (95%) | 42 (94%) | 52 (95%) | 54 (83%) | *ac35* |
| **49** |  | 41659🡪41901 | 80 | - | - | - | - | unknown [Zea mays], E = 1.6e^-12^ |
| **50** | *odv-ec43* | 41953🡪43017 | 354 | 51 (67%) | 41 (74%) | 53 (65%) | 55 (43%) | *ac109* |
| **51** |  | 43021🡪43356 | 111 | 52 (76%) | 43 (67%) | 54 (74%) | 56 (42%) | *ac108* |
| **52** | *pp31/39k* | 43470🡨44387 | 305 | 53 (62%) | 44 (63%) | 55 (61%) | 57 (27%) | *ac36* |
| **53** | *lef-11* | 44371🡨44655 | 94 | 54 (78%) | 45 (80%) | 56 (76%) | 58 (52%) | *ac37* |
|  | *hr1* | 44840-45758 |  |  |  |  |  | 5 repeats |
| **54** | *bro-b* | 46210🡪47313 | 367 | - | - | - | - | Best match: SpfrGV ORF53, E = 1e^-54^ |
| **55** |  | 47450🡨48100 | 216 | - | - | - | - |  |
| **56** |  | 48090🡪48542 | 150 | - | - | - | - | Best match: LeseNPV ORF73, E = 2e^-23^; Family g.31.1.1: Tachycitin, prob=98.9% (N-terminal copy) and 99.1% (C-terminal copy), |
| **57** |  | 48653🡨49492 | 279 | 55 (28%) | 47 (22%) | 57 (26%) | - | Best match: MacoNPV-A 90/2 ORF26, E = 9e^-20^ |
| **58** | *sod* | 49602🡨50060 | 152 | 64 (76%) | 50 (67%) | 68 (74%) | 59 (60%) | *ac31* |
| **59** |  | 50086🡨50373 | 95 | - | - | - | - | Best match: uncharacterized protein Dwil_GK16219 [Drosophila willistoni]; E = 2e^-6^; Family g.31.1.1: Tachycitin, prob=99.4% |
|  | *hr2* | 50466-51124 |  |  |  |  |  | 4 repeats |
| **60** |  | 51378🡪51953 | 191 | 70 (36%) | 55 (27%) | 71 (38%) | - |  |
| **61** |  | 52124🡪53605 | 493 | 72 (48%) | 56 (41%) | 73 (49%) | - | Homologs among entomopoxviruses |
| **62** | *bro-c* | 53749🡨55719 | 656 | - | - | - | - | Best match: Bro18 [Heliothis virescens ascovirus 3e], E = 1e^-28^ |
| **63** |  | 56152🡨56682 | 176 | - | - | - | - | Best match: AgseNPV-B ORF16, E = 3e^-28^ |
| **64** |  | 56815🡨57525 | 236 | 58 (74%) | - | 151 (47%) | - |  |
| **65** |  | 57543🡨58496 | 317 | 73 (40%) | - | 74 (43%) | - |  |
| **66** |  | 58700🡪59026 | 108 | 74 (63%) | 58 (66%) | 75 (66%) | - | *ac79* |
|  | *hr3* | 59178-59237 |  |  |  |  |  | 1 repeat |
| **67** | *bro-d* | 60793🡪61551 | 252 | 76 (48%) | 60 (50%) | 76 (47%) | - |  |
| **68** | *p74* | 61577🡪63685 | 702 | 77 (71%) | 61 (64%) | 77 (71%) | 60 (42%) | *ac138* |
| **69** |  | 63729🡪64154 | 141 | - | - | - | - | No matches |
| **70** | *p47* | 64170🡪65372 | 400 | 79 (74%) | 63 (78%) | 78 (74%) | 68 (56%) | *ac40* |
| **71** | Nudix; ADP-ribose pyrophosphatase | 65461🡪66135 | 224 | 82 (85%) | 64 (85%) | 79 (83%) | 69 (69%) | *ac38* |
| **72** | *p24* | 66165🡪66665 | 166 | 83 (70%) | 65 (67%) | 80 (70%) | 71 (48%) | *ac129* |
| **73** | *38.7k* | 66691🡨67206 | 171 | 84 (48%) | 66 (43%) | 81 (49%) | 73 (28%) | *ac13* |
| **74** | *lef-1* | 67213🡨67929 | 238 | 85 (78%) | 67 (71%) | 82 (76%) | 74 (49%) | *ac14* |
| **75** | *p10* | 68002🡪68502 | 166 | 86 (51%) | 68 (57%) | 83 (57%) | - |  |
| **76** | *pif-1* | 68517🡪70145 | 542 | 87 (68%) | 69 (58%) | 84 (68%) | 75 (46%) | *ac119* |
| **77** | *fgf-1* | 70188🡨70910 | 240 | 88 (45%) | 70 (45%) | 85 (39%) | 76 (28%) |  |
| **78** |  | 70961🡨71317 | 118 | 89 (43%) | - | 86 (44%) | - |  |
| **79** |  | 71369🡪71851 | 160 | 90 (30%) | 71 (46%) | 87 (28%) | 79 (34%) | *ac150* |
| **80** |  | 71857🡪72342 | 161 | 90 (59%) | 72 (40%) | 87 (60%) | 79 (33%) | *ac150* |
| **81** | *lef-6* | 72348🡨72632 | 94 | 91 (57%) | 73 (65%) | 88 (57%) | 80 (46%) | *ac28* |
| **82** | *dbp* | 72684🡨73529 | 281 | 92 (56%) | 74 (43%) | 89 (56%) | - | *ac25* |
| **83** |  | 73584🡨73805 | 73 | 93 (61%) | - | - | - |  |
| **84** |  | 73778🡨74497 | 239 | 94 (52%) | 76 (45%) | 90 (51%) | - |  |
| **85** | *p45* | 74496🡪75614 | 372 | 95 (82%) | 75 (82%) | 91 (82%) | 83 (54%) | *ac103* |
| **86** | *p12* | 75617🡪75964 | 115 | 96 (59%) | 77 (67%) | 92 (61%) | 84 (41%) | *ac102* |
| **87** | *p40* (*bv/odv-c42*) | 76018🡪77139 | 373 | 97 (76%) | 78 (80%) | 93 (75%) | 85 (52%) | *ac101* |
| **88** | *p6.9* | 77158🡪77340 | 60 | 98 (79%) | 79 (77%) | 94 (79%) | 86 (63%) | *ac100* |
| **89** | *lef-5* | 77431🡨78204 | 257 | 99 (74%) | 81 (74%) | 95 (73%) | 87 (55%) | *ac99* |
| **90** | *38k* | 78118🡪79038 | 306 | 100 (67%) | 80 (73%) | 96 (67%) | 88 (48%) | *ac98* |
| **91** | *pif-4* | 79060🡨79533 | 157 | 101 (82%) | 83 (78%) | 97 (79%) | 89 (51%) | *ac96* |
| **92** | *helicase-1* | 79532🡪83005 | 1157 | 102 (78%) | 82 (76%) | 98 (78%) | 90 (38%) | *ac95* |
| **93** | *odv-e25* | 83060🡨83716 | 218 | 103 (84%) | 85 (79%) | 99 (85%) | 91 (68%) | *ac94* |
| **94** | *p18* | 83765🡨84241 | 158 | 104 (66%) | 87 (76%) | 100 (67%) | 92 (44%) | *ac93* |
| **95** | *p33* | 84322🡪85077 | 251 | 105 (82%) | 86 (73%) | 101 (82%) | 93 (54%) | *ac92* |
| **96** | *chaB* | 85087🡨85344 | 85 | 106 (82%) | 88 (90%) | 102 (79%) | - | *ac60* |
| **97** |  | 85384🡨85641 | 85 | - | - | - | - |  |
| **98** | *dut* | 85780🡪86217 | 145 | - | - | - | - | deoxyuridine 5'-triphosphate nucleotidohydrolase [Microplitis demolitor], E = 4e^-59^ |
| **99** |  | 86300🡨87280 | 326 | 73 (29%) | - | 74 (31%) | - |  |
| **100** | *lef-4* | 87342🡨88694 | 450 | 114 (63%) | 91 (67%) | 110 (63%) | 95 (44%) | *ac90* |
| **101** | *vp39* | 88745🡪89719 | 324 | 115 (77%) | 92 (75%) | 111 (73%) | 96 (39%) | *ac89* |
| **102** | *odv-ec27* | 89790🡪90656 | 288 | 116 (81%) | 93 (80%) | 112 (82%) | 97 (46%) | *ac144* |
| **103** |  | 91047🡨92177 | 376 | 117 (54%) | 94 (57%) | 113 (54%) | 99 (29%) |  |
| **104** | *bro-e* | 92445🡨93542 | 365 | 118 (68%) | 95 (67%) | 114 (69%) | - |  |
| **105** |  | 93683🡪94660 | 325 | 119 (45%) | 96 (49%) | 115 (46%) | - |  |
| **106** |  | 94794🡪95183 | 129 | 120 (66%) | 97 (67%) | 116 (66%) | 100 (50%) |  |
| **107** |  | 95223🡨95843 | 206 | 121 (39%) | - | 117 (39%) | - |  |
| **108** | *vp91* | 95896🡨98007 | 703 | 122 (58%) | 101 (60%) | 118 (58%) | 101 (34%) | *ac83* |
| **109** | *tlp-20* | 97973🡪98479 | 168 | 123 (63%) | 100 (63%) | 119 (60%) | 102 (NSS) | *ac82* |
| **110** |  | 98499🡪99071 | 190 | 124 (80%) | 102 (86%) | 120 (82%) | 103 (60%) | *ac81* |
| **111** | *gp41* | 99110🡪99991 | 293 | 125 (71%) | 103 (78%) | 121 (73%) | 104 (50%) | *ac80* |
| **112** |  | 100055🡪100369 | 104 | 126 (52%) | 104 (57%) | 122 (54%) | 105 (NSS) | *ac78* |
| **113** | *vlf-1* | 100347🡪101477 | 376 | 127 (75%) | 105 (83%) | 123 (74%) | 106 (54%) | *ac77* |
| **114** |  | 101482🡨102024 | 180 | 128 (64%) | 107 (59%) | 124 (64%) | - |  |
| **115** |  | 102062🡪102319 | 85 | 129 (88%) | 106 (92%) | 125 (86%) | 107 (60%) | *ac76* |
| **116** |  | 102347🡪102787 | 146 | 130 (54%) | 108 (53%) | 126 (56%) | 108 (NSS) | *ac75* |
| **117** | *dnapol* | 102889🡨106002 | 1037 | 138 (75%) | 109 (76%) | 132 (78%) | 111 (53%) | *ac65* |
| **118** | *desmoplakin* | 106001🡪107908 | 635 | 139 (48%) | 110 (49%) | 133 (47%) | 112 (48%) | *ac66* |
| **119** | *lef-3* | 108038🡨109087 | 349 | 140 (48%) | 112 (48%) | 134 (48%) | 113 (26%) | *ac67* |
| **120** | *pif-6* | 109056🡪109472 | 138 | 141 (79%) | 111 (65%) | 135 (78%) | 114 (42%) | *ac68* |
| **121** |  | 109498🡪110040 | 180 | 142 (43%) | 113 (36%) | 136 (42%) | - |  |
| **122** | *iap-5* | 110088🡪110897 | 269 | 143 (53%) | 114 (61%) | 137 (52%) | 116 (29%) |  |
| **123** | *lef-9* | 110962🡪112455 | 497 | 145 (81%) | 115 (83%) | 139 (82%) | 117 (63%) | *ac62* |
| **124** | *fp25k* | 112512🡪112958 | 148 | 146 (83%) | 116 (87%) | 140 (82%) | 118 (51%) | *ac61* |
| **125** | *ligase* | 112960🡨114546 | 528 | 148 (71%) | 117 (72%) | 141 (71%) | 120 (44%) |  |
| **126** |  | 114716🡪114928 | 70 | 149 (62%) | 118 (71%) | 142 (76%) | - |  |
| **127** |  | 114922🡪115200 | 92 | 150 (81%) | 119 (66%) | 143 (81%) | - |  |
| **128** | *fgf-2* | 115238🡨116377 | 379 | 151 (46%) | 120 (61%) | 144 (46%) | 123 (27%) | *ac32* |
| **129** | *alk-exo* | 116501🡪117703 | 400 | 152 (61%) | 121 (65%) | 145 (59%) | 125 (44%) | *ac133* |
| **130** | *helicase-2* | 117713🡪119095 | 460 | 153 (71%) | 122 (67%) | 146 (69%) | 126 (48%) |  |
| **131** |  | 119211🡪120203 | 330 | 154 (61%) | 123 (57%) | 147 (60%) | - | *ac112/113* |
| **132** | *lef-8* | 120237🡨122810 | 857 | 155 (79%) | 124 (83%) | 148 (79%) | 131 (60%) | *ac50* |
| **133** | *odv-e66* | 122913🡨124919 | 668 | 156 (79%) | 126 (73%) | 149 (78%) | 37 (43%) | *ac46* |
|  | *hr4* | 124993-125388 |  |  |  |  |  | 3 repeats |
| **134** | *enhancin-1* | 125418🡨127973 | 851 | 157 (47%) | - | 150 (48%) | - |  |
| **135** | *enhancin-2* | 127981🡨130605 | 874 | 159 (35%) | 127 (45%) | 152 (55%) | - |  |
| **136** |  | 130882🡨131112 | 76 | 163 (63%) | - | 160 (54%) | - | Best match: LeseNPV ORF48, E = 1e^-27^ |
| **137** |  | 131194🡪132540 | 448 | 164 (51%) | 128 (55%) | 161 (52%) | - | Ascovirus and entomopoxvirus homologs; pfam13930 Endonuclea_NS_2 (DNA/RNA non-specific endonuclease), prob=99.8% |
| **138** |  | 132607🡨133179 | 190 | 165 (40%) | 130 (42%) | 162 (39%) | - |  |
|  | *hr5* | 133338-133394 |  |  |  |  |  | 1 repeat |
| **139** |  | 133433🡨133789 | 118 | 168 (56%) | - | - | - |  |
| **140** | *enhancin-3* | 134020🡨136605 | 861 | 170 (55%) | 132 (43%) | 166 (54%) | - |  |
| **141** |  | 137013🡪137381 | 122 | 171 (55%) | 133 (37%) | 167 (57%) | - |  |
|  | *hr6* | 137650-137898 |  |  |  |  |  | 2 repeats |
| **142** |  | 137941🡨138126 | 62 | 172 (38%) | - | 170 (44%) | - |  |
| **143** | U-box/RING-like domain | 138113🡪138532 | 139 | 173 (56%) | 135 (69%) | 171 (57%) | 134 (48%) | *ac53* |
| **144** |  | 138522🡨139748 | 408 | 174 (50%) | 136 (54%) | 172 (49%) | - |  |
| **145** |  | 139761🡨139964 | 67 | 175 (72%) | 138 (73%) | 173 (70%) | - |  |
| **146** | *lef-10* | 139849🡪140157 | 102 | 176 (78%) | 137 (75%) | 174 (76%) | - | *ac53a* |
| **147** | *vp1054* | 140036🡪141007 | 323 | 177 (78%) | 139 (73%) | 175 (76%) | 138 (48%) | *ac54* |
| **148** |  | 141099🡪141278 | 59 | 178 (72%) | 140 (66%) | 176 (70%) | - |  |
| **149** |  | 141352🡪141663 | 103 | 179 (42%) | 141 (41%) | 177 (41%) | - |  |
| **150** | *fgf-3* | 141693🡪142664 | 323 | 180 (52%) | 142 (62%) | 178 (50%) | 140 (25%) |  |
| **151** |  | 142746🡪143336 | 196 | 181 (45%) | 143b (31%) | 179 (46%) | - |  |
| **152** | *me53* | 143403🡪144299 | 298 | 182 (63%) | 144 (64%) | 180 (66%) | 143 (32%) | *ac139* |
| **153** |  | 144305🡪144628 | 107 | 183 (63%) | 145 (73%) | 181 (65%) | - |  |

^a^NSS: No significant sequence similarity detected by blastp

^b^Some pairs of ORFs in the SpfrGV-VG008 genome were numbered in the opposite order in which they occur in the genome sequence. These ORFs are : 2 and 3; 6 and 7; 41 and 42; 75 and 76; 80 and 81; 82 and 83; 86 and 87; 100 and 101; 106 and 107; 111 and 112; and 137 and 138

^c^E-values are from blastp queries; true-positive probabilities (prob) are from queries with HHpred.
